# Distinct functional and molecular profiles between physiological and pathological atrial enlargement offer potential new therapeutic opportunities for atrial fibrillation

**DOI:** 10.1042/CS20240178

**Published:** 2024-07-30

**Authors:** Yi Ching Chen, Seka Wijekoon, Aya Matsumoto, Jieting Luo, Helen Kiriazis, Emma Masterman, Gunes Yildiz, Jonathon Cross, Adam C. Parslow, Roger Chooi, Junichi Sadoshima, David W. Greening, Kate L. Weeks, Julie R. McMullen

**Affiliations:** 1 Baker Heart and Diabetes Institute, Melbourne, Victoria, Australia; 2 Department of Diabetes, Central Clinical School, Monash University, Clayton, Victoria, Australia; 3 Baker Department of Cardiometabolic Health, The University of Melbourne, Melbourne, Victoria, Australia; 4 Baker Department of Cardiovascular Research, Translation and Implementation, La Trobe University, Melbourne, Victoria, Australia; 5 Department of Cell Biology and Molecular Medicine, Rutgers New Jersey Medical School, NJ, U.S.A.; 6 Department of Anatomy and Physiology, University of Melbourne, Melbourne, Victoria, Australia; 7 Monash Alfred Baker Centre for Cardiovascular Research, Monash University, Melbourne, Victoria, Australia

**Keywords:** biochemical techniques and resources, cardiac arrhythmia, drug discovery and design, myocardium, proteomics

## Abstract

Atrial fibrillation (AF) remains challenging to prevent and treat. A key feature of AF is atrial enlargement. However, not all atrial enlargement progresses to AF. Atrial enlargement in response to physiological stimuli such as exercise is typically benign and reversible. Understanding the differences in atrial function and molecular profile underpinning pathological and physiological atrial remodelling will be critical for identifying new strategies for AF. The discovery of molecular mechanisms responsible for pathological and physiological ventricular hypertrophy has uncovered new drug targets for heart failure. Studies in the atria have been limited in comparison. Here, we characterised mouse atria from (1) a pathological model (cardiomyocyte-specific transgenic (Tg) that develops dilated cardiomyopathy [DCM] and AF due to reduced protective signalling [PI3K]; DCM-dnPI3K), and (2) a physiological model (cardiomyocyte-specific Tg with an enlarged heart due to increased insulin-like growth factor 1 receptor; IGF1R). Both models presented with an increase in atrial mass, but displayed distinct functional, cellular, histological and molecular phenotypes. Atrial enlargement in the DCM-dnPI3K Tg, but not IGF1R Tg, was associated with atrial dysfunction, fibrosis and a heart failure gene expression pattern. Atrial proteomics identified protein networks related to cardiac contractility, sarcomere assembly, metabolism, mitochondria, and extracellular matrix which were differentially regulated in the models; many co-identified in atrial proteomics data sets from human AF. In summary, physiological and pathological atrial enlargement are associated with distinct features, and the proteomic dataset provides a resource to study potential new regulators of atrial biology and function, drug targets and biomarkers for AF.

## Introduction

Atrial fibrillation (AF) is the most common cardiac arrhythmia worldwide (∼37 million) and is expected to continue rising in prevalence due to an aging population and increasing rates of obesity and diabetes [1]. Patients with AF are at a notably heightened risk of developing heart failure (HF), stroke and neurological decline. Catheter ablation and/or physical activity have been successful in some patients [2], but AF recurrence remains common, particularly in those with persistent AF [3]. Most current drugs have limited or no efficacy, and potentially dangerous side effects [4–7]. Thus, there is a compelling need for further research to identify potential new therapeutic targets that could lead to more effective interventions for AF.

Atrial enlargement in response to a cardiac stress stimulus, such as hypertension, predisposes both experimental animals and humans to AF. However, atrial enlargement can also occur in response to physiological stimuli such as exercise and pregnancy [8]. This type of enlargement is not typically associated with AF, and is reversible [9]. Understanding the different mechanisms underlying physiological and pathological atrial enlargement/atrial myopathy could provide opportunities to identify new treatments or biomarkers for AF. Genetic mouse models have been invaluable for assessing the key molecular mechanisms responsible for pathological and physiological ventricular hypertrophy, resulting in the identification of new drug targets for HF [10,11]. To date, comparable studies in the mouse atria have been limited.

The insulin-like growth factor 1 receptor (IGF1R)-phosphoinositide 3-kinase (PI3K) signalling pathway has been shown to play a critical role in regulating physiological postnatal cardiac growth and exercise-induced cardiac hypertrophy in adult mice [12,13]. Cardiomyocyte-specific transgenic (Tg) mice with increased IGF1R and/or PI3K signalling have enlarged ventricles with preserved or enhanced cardiac function [12,14]. In contrast, cardiomyocyte-specific Tg mice expressing a dominant negative PI3K mutant to decrease PI3K activity had smaller hearts under basal conditions [14], and displayed a blunted hypertrophic response to swim exercise training [13] and IGF1R-induced physiological hypertrophy [12]. Furthermore, PI3K is critical for protecting the heart in cardiac stress settings. In a setting of dilated cardiomyopathy (DCM), a reduction in PI3K led to accelerated HF and intermittent AF in mice [15,16]. In contrast, cardiomyocyte-specific Tg mice with increased IGF1R and/or PI3K are protected in cardiac stress settings [12,17–20].

Previous studies characterising the DCM model with reduced PI3K (DCM-dnPI3K) and the IGF1R Tg model [12,15,16], focused primarily on the ventricle rather than the atria. This was largely due to limitations in accurately assessing atrial function in the very small mouse atria (i.e. ∼2–5 mg atrium vs. 100 mg ventricle for a male 25–30 g mouse). Significant improvements in imaging technology and the ability to profile very small amounts of tissue now allow studies in mouse atria to be performed. Atrial and ventricular myocytes possess distinct functional roles, with atrial myocytes exhibiting unique stretch-sensing, electrical, contractile, and secretory properties attributed to specific molecular profiles [21]. Thus, understanding the specific changes in atrial function and molecular profile in settings of health and disease will be critical for understanding the mechanisms underlying pathological atrial enlargement and development of AF.

In the present study, we conducted a thorough characterisation of the atria and atrial myocytes from two cardiomyocyte-specific transgenic mouse models: IGF1R Tg mice which display ventricular hypertrophy accompanied by enhanced systolic ventricular function, no evidence of arrhythmia, and enlarged atria [12,16], and DCM-dnPI3K Tg mice which display ventricular dilatation, reduced systolic ventricular function, arrhythmia including intermittent AF, enlarged atria and lung congestion [15,16]. We hypothesised that atrial function would be depressed in the DCM-dnPI3K Tg model but not the IGF1R Tg model, and this would be associated with differences in atrial myocyte dimensions, fibrosis and molecular profile. An improved understanding of physiological and pathological atrial enlargement is expected to provide new opportunities to prevent or treat pathological atrial enlargement and AF.

## Methods

### Experimental animals

Animal experimentation in this study was conducted in accordance with the guidelines and approval of the Alfred Research Alliance (ARA) Animal Ethics Committee, Vic, Australia (ethics approval numbers, E/3587/2021/B and E/1859/2018/B). All animal work was performed in PAC (People and Cures): 75 Commercial Road, Melbourne, Victoria 3004, Australia. The animals were subjected to a 12-h light cycle and provided with standard chow and water.

The study used two previously described cardiomyocyte specific Tg mouse models which had been generated using the α-myosin heavy chain promoter. The physiological model was produced by breeding hemizygous male cardiomyocyte-specific IGF1R Tg mice with female non-transgenic (Ntg) mice [12], and the pathological model was produced by breeding hemizygous male cardiomyocyte-specific mammalian sterile 20-like kinase 1 (Mst1) Tg mice with hemizygous female cardiomyocyte-specific dnPI3K Tg mice [14,15,22]. The Mst1 Tg model develops DCM due to increased activation of caspases and apoptosis, and the double Tg (Mst1-dnPI3K Tg) develops more severe DCM and intermittent AF [15]. These mice are subsequently referred to as DCM-dnPI3K Tg. The majority of the characterisation in the IGF1R Tg and DCM-dnPI3K Tg was performed in 20-week-old mice because based on our previous work, both models display atrial enlargement by this age [16], and increasing numbers of the DCM-dnPI3K Tg model can die suddenly from arrhythmia or heart failure at >20 weeks of age [15]. Mice were genotyped by performing PCR on genomic DNA extracted from tail tissue. Primer sequences for detecting the IGF1R, Mst1 and dnPI3K transgenes are listed in Supplementary Table S1.

### Experimental cohorts

Cohort 1: Male and female IGF1R Tg, DCM-dnPI3K Tg and Ntg littermate controls at ∼20 weeks of age were used for assessment of atrial function, morphological assessment and gene expression.

Cohort 2: Female IGF1R Tg, DCM-dnPI3K Tg and Ntg littermate controls at ∼20 weeks of age were used for heart collection and atrial fibrosis assessment.

Cohort 3: Female IGF1R Tg, DCM-dnPI3K Tg and Ntg littermate controls at ∼20 weeks of age were used for isolation of atrial and ventricular myocytes.

Cohort 4: Female IGF1R Tg, DCM-dnPI3K Tg and littermate controls at ∼8 weeks age were used for left atrium (LA) proteomic analysis (Western blotting and comprehensive proteomic profiling by mass spectrometry).

Cohort 5: Female IGF1R Tg, DCM-dnPI3K Tg and littermate controls at ∼8 weeks age were used for assessment of atrial function, morphological assessment and gene expression.

Details of cohorts and any exclusions are outlined in Supplementary Figure S1.

### Atrial function by echocardiography

Atrial structure and function were assessed in anesthetised mice (isoflurane: 3.5–4% induction, 1.5–2% maintenance) using a Vevo 2100 Ultrasound Machine (VisualSonics) with a 40-MHz transducer. LA, LA and appendage (LA+APP), and right atria (RA) dimensions and function were assessed using a 4-chamber view, adjusted to capture the LA appendage. Images were captured and measures made at 3 different points in the beat cycle using Vevo LAB v5.7.1 software. Volume-derived measures of atrial function were calculated using the equations in the Table below following nomenclature in Baysan et al. [23]. LA volume (LAV) pre atrial contraction (pre-A): (LAV_preA_) was measured at p-wave onset, LAV minimum (LAV_min_) measured at the R-wave, LAV maximum (LAV_max_) measured one frame prior to the mitral valve opening. LA volumes were measured using the Vevo LAB trace tool. The RA and LA+LAA data are presented as areas.

**Table:** Measures of LA, LA+APP, and RA function

LA reservoir function	LA total emptying volume: LAV_max_-LAV_min_
	LA total emptying fraction: (LAV_max_-LAV_min_)/LAV_max_
	LA ejection fraction (%): LA total emptying fraction x 100
LA conduit function	LA passive emptying volume: LAV_max_-LAV_preA_
	LA passive emptying fraction: (LAV_max_-LAV_preA_)/LAV_max_
LA booster pump function	LA active emptying volume: LAV_preA_-LAV_min_
	LA active emptying fraction: (LAV_preA_-LAV_min_)/LAV_preA_
LAA+APP function	LA+APP total emptying fraction: (LA+APP Area_max_-LA+APP Area_min_)/LA+APP Area_max_
RA function	RA total emptying fraction: (RA Area_max_-RA Area_min_)/RA Area_max_

All image acquisition and data analyses were performed blinded, and measures validated by an independent investigator [24].

### Atrial and ventricular myocyte isolation

Atrial and ventricular myocytes were isolated as described [25], with some modifications. Mice were injected with heparin (1 unit/g body weight) 15 min before euthanasia (pentobarbitone 300 mg/kg i.p. followed by cervical dislocation). The heart was explanted and immediately cannulated for perfusion with filtered perfusion medium (5.4 mM potassium chloride, 3.5 mM magnesium sulfate, 0.05 mM sodium pyruvate, 20 mM sodium bicarbonate, 11 mM glucose, 20 mM HEPES, 23.5 mM sodium glutamate, 4.87 mM sodium acetate, 56 μM phenol red, 10 mM 2,3-butanedione monoxime, 5 mM creatine, 30 mM taurine, 0.1 IU ml^−1^ insulin, gassed with 95% O_2_ and 5% CO_2_, pH 7.25) and collagenase II (3 mg ml^−1^) for ∼15 min (flow rate 2–3 ml min^−1^). Atria were separated, minced, and gently teased apart using fine-tip surgical scissors and forceps. After gentle trituration with a glass pipette, stopping buffer (perfusion medium supplemented with 10% (v/v) FBS and 125 μM CaCl_2_) was added, cells sedimented by gravity for 10 min and centrifuged at 20 g for 5 min. The pellet was resuspended in filtered plating medium (DMEM containing 0.04% [v/v] FBS, 1× insulin-transferrin-selenium, 0.1 μM corticosterone), plated on laminin-coated imaging dishes (Ibidi μ-Slide 8-well) and incubated at 37°C for 1 h. The media (containing unadhered cells) was aspirated and replaced with plating medium supplemented with 10 mM 2,3-butanedione monoxime prior to imaging. Ventricular myocytes underwent additional washes before plating in 35 mm Ibidi imaging dishes also coated with laminin. Images of atrial myocytes were acquired using a 20× LUCPlanFLN (NA 0.70) objective lens on an Olympus IX71 inverted microscope with a DP80 camera. Images of ventricular myocytes were acquired using a 4× LUCPlanFLN (NA 0.13) objective lens on an Olympus IX71 microscope with a DP25 camera. All image acquisition was performed in a blinded manner with respect to genotype.

### Assessment of atrial and ventricular myocyte dimensions

The dimensions (length, width, and area) of atrial and ventricular myocytes were analysed using ImageJ software (version 2.1.0, NIH) [26] in a blinded manner with respect to genotype. For atrial myocytes, the freehand selection tool was used to draw around the edge of each myocyte, and the area, maximum Feret’s diameter (length), and minimum Feret’s diameter (width) were measured. Myocytes were excluded from the analysis if they were round or starting to ball up (indicating dead or dying myocytes), not linear (as this would lead to inaccurate measurement of cell length), split or attached to another cell, partially covered by debris or another cell, or if they were out of focus or not fully adhered to the cell culture plate. For assessment of ventricular myocyte dimensions, grayscale images of the myocytes were converted to binary images. The ‘fill hole’ function was applied, and all objects representing single myocytes (excluding those meeting the criteria below) were selected using the wand tool. Area, maximum Feret’s diameter (length), and minimum Feret’s diameter (width) were measured as described for atrial myocytes. Myocytes were excluded from the analysis if the binary overlay of the cell contained holes or was incomplete, the cell was round or starting to ball up, the cell was split or attached to another cell, the cell was touching another cell or debris (as the cell boundary could not be resolved once the binary mask had been applied), or if the cell was out of focus or not fully adhered to the cell culture plate.

### Tissue collection

Mice were humanely euthanised with pentobarbital (300–400 mg/kg body weight i.p.) followed by cervical dislocation. Tissues, including mouse atria and ventricles were rinsed with PBS, dried with gauze, weighed, snap-frozen in liquid nitrogen, and stored at −80°C for subsequent extractions of proteins and/or RNA. To measure the tibia length (TL), a hind leg was collected and the muscle and fat were digested in 1 M sodium hydroxide at 37 °C for ∼ 6–8 h, followed by measuring with a vernier caliper.

### Protein extraction and Western blotting

Frozen tissues were homogenised (PRO Scientific, Model Pro 200) in ice-cold lysis buffer consisting of 10% glycerol, 137 mM NaCl, 20 mM Tris/HCl (pH 7.4), 20 mM sodium fluoride, 10 mM EDTA, 1 mM EGTA, 1 mM sodium pyrophosphate, 1 mM vanadate, 1 mM phenylmethylsulfonyl fluoride, 4 μg ml^−1^ pepstatin, 4 μg ml^−1^ aprotinin, and 4 μg ml^−1^ leupeptin; followed by the addition of Igepal CA-630 (Sigma) to a final concentration of 1% (v/v). After vortexing, the sample incubated on ice for 15 min to lyse cellular membranes, followed by centrifugation for 15 min at 16,560 g at 4°C. The supernatant was collected, and protein concentration determined using the Bradford assay (2.1.7.1, Bio-Rad, 50000-0006).

For Western blot analysis, samples containing 30 μg of protein in sample buffer were denatured (95°C for 5 min). Samples were loaded onto 10% SDS-PAGE gels alongside 20 μl of Kaleidoscope marker (BioRad, 1610374), separated at 150 V for 40–60 min, then transferred to PVDF membranes by wet transfer (9V at 4°C overnight). Membranes were baked for 10 min at 65°C for storage. Membranes were activated (methanol for 1 min, double distilled water for 2 min, and TBST for 5 min), blocked in 5% milk/TBST for 1 h then incubated in primary antibody at 4°C overnight. The following concentrations of primary antibodies were used: 1:1000 IGF1Rβ (Cell Signaling, #3027), 1:1000 PI3K (p110α, Cell Signaling, #4249), and 1:5000 GAPDH (Santa Cruz, sc-32233). The next day, membranes were washed four times for 5 minutes each in TBST, followed by the addition of the secondary antibody (1.5 h at room temperature), four 5 min TBST washes, and exposure to chemiluminescent reagent (Amersham HyperfilmTM ECL, Crown) for HRP-conjugated secondary antibody detection. Membrane bands were imaged using a Syngene GBox Image Developer machine, and the quantification of bands was performed using GeneTools software v4.4.

### RNA extraction and qPCR analysis

LA was homogenised in Trizol (500 μl per 2 mg of tissue) using a Bio-gen series Pro 200 Pro scientific homogeniser and kept on ice. After 5 min of incubation at room temperature, 100 μl of chloroform was added to each sample, agitated vigorously, and centrifuged for 10 min (18,630 ***g*** at 4°C). The upper phase was collected, 1.5× volume of 100% ethanol added, sample run through a column (Qiagen RNeasy Plus Mini kit; Cat. No. / ID: 74104), and the RNA was DNase-treated and washed using the Qiagen RNeasy kit as per the manufacturer’s instructions. Extracted RNA was stored at −80°C until required, and RNA concentrations determined using a nanophotometer (Lab gear Australia, IMPLEN).

RNA (2 μg) was subjected to reverse transcription using the High Capacity cDNA Reverse Transcription Kit (Life Technologies, Thermo Fisher Scientific). The resulting cDNA was used for qPCR using either TaqMan Gene Expression Assays or SYBR Green chemistry and amplified on an Applied Biosystems 7500, Quant Studio 6 or 7 Flex real-time PCR instrument (primer details are provided in Supplementary Table S2). For TaqMan Gene Expression Assays, gene expression was normalised to hypoxanthine phosphoribosyltransferase 1 (*Hprt1*) using the 2^−ΔΔCt^ method of quantification. For SYBR Green qPCR, a standard curve was generated using serial dilutions of cDNA, and real-time PCR efficiencies were calculated using the equation *E* = 10^[–1/slope]^, where the slope represents the regression of the standard curve. Gene expression was normalised to *Hprt1* and quantified using the comparative *C*t method (an efficiency corrected calculation model). Samples were run in triplicate. Prior to analysis, it was established that only SD<0.5 be used. Duplicates were used if exclusion of one value ensured the SD<0.5.

### Proteomic sample preparation for mass spectrometry

Mouse LA (physiological model: IGF1R and Ntg littermates [labelled NTG2], pathological model: DCM-dnPI3K [HF+AF] and Ntg littermates [labelled NTG4]), were extracted and lysed on ice in lysis buffer (8 M urea in 50 mM HEPES pH 8.0 with Halt™ Protease/Phosphatase Inhibitor Cocktail (#78442, Thermo Fisher Scientific)) and extracted by pulse tip-probe sonication on ice and quantified by microBCA (#23235, Life Technologies). All samples quantified by microBCA (Thermo Fisher Scientific), normalised (10 µg) and subsequently reduced, alkylated and solid-phase interaction proteomic sample preparation performed as described [27]. Briefly, protein samples were mixed with Sera‐Mag Speed Beads (#45152105050250 and #65152105050250, GE LifeScience) at a 10:1 beads-to-protein ratio, with protein-bound-beads reconstituted and exposed to trypsin (V5113, Promega) and Lys-C (121-05063, FUJIFILM Wako Pure Chemical Corporation) at 1:50 and 1:100 enzyme-to-substrate ratio overnight at 37°C with shaking. Peptide digests were collected from the supernatant and acidified with formic acid to a final 2% (v/v) concentration before vacuum lyophilised and reconstituted in 0.07% (v/v) trifluoroacetic acid in MS-grade water. Subsequently, peptides were quantified by Fluorometric Peptide Assay (#23290, Thermo Fisher Scientific) as per manufacturer’s instructions.

### Proteomics: NanoLC and mass spectrometry

Spectra was acquired in data independent acquisition on an Q Exactive HF-X benchtop Orbitrap mass spectrometer coupled to an UltiMate™ NCS-3500RS nano-HPLC (Thermo Fisher Scientific) as described [28]. Briefly, peptides were loaded (Acclaim PepMap100 C18 3 µm beads with 100 Å pore-size, Thermo Fisher Scientific) and separated (1.9-µm particle size C18, 0.075 × 250 mm, Nikkyo Technos Co. Ltd) with a gradient of 2–28% acetonitrile containing 0.1% formic acid over 95 min followed by 28–80% from 95–98 min at 300 nl min^−1^ at 55°C (butterfly portfolio heater, Phoenix S&T). A MS1 full scan was set to 60,000 resolution, 3e6 AGC target and maximum IT of 50 ms in 350–1100 *m*/*z* scan range. MS2 was set to 15,000 resolution, 1e6 AGC target and 27 ms maximum IT. A total of 63 scan windows with staggered 12 *m*/*z* isolation window from 350 to 1100 *m*/*z* were applied with 28% normalised collision energy [28]. Data was acquired using Xcalibur software v4.5 (Thermo Fisher Scientific).

MS-based proteomics data are deposited to the ProteomeXchange Consortium via the MASSive partner repository and available via MASSive with identifier (MSV000093605).

### MS data processing and analysis

Identification and quantification of proteins was performed using DIA-NN neural network and interference correction (v1.8) with mass spectra searched against *Mus musculus* (mouse) reference proteome (55,398) supplemented with common contaminants. Spectral libraries were predicted using the deep learning algorithm employed by in DIA-NN [29] with Trypsin/P, allowing up to 1 missed cleavage. The precursor change range was set to 1–4, and the *m*/*z* precursor range was set to 300–1800 for peptides consisting of 7–30 amino acids with N-term methionine excision and cysteine carbamidomethylation enabled as a fixed modification with 0 maximum number of variable modifications. The mass spectra were analysed using default settings with a false discovery rate (FDR) of 1% for precursor identifications and match between runs (MBR) enabled for replicates, single-parse mode enabled. We further applied data quality inclusion within each group at a minimum 50% protein group quantitation. Perseus (v2.0.7.0) was applied for downstream data processing and statistical analyses, with scatter plots generated using GraphPad Prism (v8.0.1) or Microsoft Excel. Stringent data quality inclusion was applied with 100% protein group quantification for proteins within a group (i.e., 3/3 replicates in one or more groups). Protein intensities were log2 transformed and normalised using quantile normalisation. Hierarchical clustering was performed in Perseus using Euclidian distance and average linkage clustering, with missing values imputed from normal distribution (width 0.3, downshift 1.8). Proteins were subjected to PCA and unpaired Student’s *t*-test. For Gene Ontology functional enrichment and network/pathway analyses, g:Profiler database [30] was employed (significance *P*<0.05). Multi-parameter (non-parametric) differential expression analysis (ANOVA, *P*<0.05) was performed in Perseus, with increased (positive) and decreased (negative) expression profiles (FC) for individual proteins relative to NTG2/4 baseline (average, log2 intensity values). *Z*-scores for proteins of interest for which *P*<0.05 in the ANOVA analysis were graphed in GraphPad Prism (v8.0.1), and unpaired *t*-tests performed to compare experimental groups (i.e., IGF1R, DCM-dnPI3K) with their respective Ntg control group. Comparative analysis of proteome studies (specifically differential proteome subsets) from atria of patients with AF and atria from human healthy heart was performed [31–33]. Selection criteria of differentially expressed proteins from the human data sets was based on the experimental design of the original papers and analyses. For study (i) human atrial fibrillation tissue: *P*<0.05 and enrichment in atria versus ventricle (ratio between each tissue source) [31], study (ii) human atrial fibrillation tissue (protein/gene): based on enrichment (*Q*<0.1, fold change>1.5, *P*<0.05, relative to non-AF) [32], and study (iii) left atria (LA) from healthy human heart and patients with AF: FDR<0.05 [33].

### Histology

LA were fixed in 4% paraformaldehyde at 4°C overnight then dehydrated and embedded in paraffin by the Monash pathology department (Melbourne VIC, Australia). Masson’s trichrome staining was used to stain deparaffinised 4 μm sections. Images of the LA were captured using a 4× UPlanFLN (NA 0.13) objective lens on an Olympus BX43 upright microscope with a DP28 camera. Collagen content was quantified by counting the number of blue pixels using an automated script in Image-Pro Analyzer (Version 7.0). The percentage of fibrosis was calculated by dividing the total area of collagen by the total area of the LA and then multiplying by 100%.

### Statistical analysis

Statistical analyses were performed using GraphPad Prism software v.8.1.2. Data are presented as mean ± SEM. The D’Agostino and Pearson normality test was used to check normality before applying statistical tests. An unpaired *t-*test was used for parametric data, and the Mann–Whitney test was used for non-parametric data, for comparison of two groups. A *P*-value of less than 0.05 was considered significant. Relative units are expressed as fold change, with the control group normalised to 1.

## Results

### Both physiological and pathological ventricular remodelling are associated with atrial enlargement

To investigate the atrial phenotype of cardiomyocyte-specific Tg mice with physiological cardiac hypertrophy induced by overexpression of the IGF1R (IGF1R Tg), or pathological cardiac remodelling induced by expression of dnPI3K to reduce PI3K activity in a mouse model of DCM (DCM-dnPI3K Tg), we first confirmed the presence of ventricular hypertrophy and remodelling in our experimental cohort. In the physiological model, both female and male IGF1R groups displayed an increase in mean heart weight, left ventricular (LV) weight and right ventricular (RV) weight compared with the Ntg control groups, indicative of LV and RV hypertrophy (Tables 1 and 2). In the pathological model, female DCM-dnPI3K mice displayed an increase in LV and RV weight versus Ntg (Table 1), while LV weight was lower and RV weight not different in male DCM-dnPI3K mice versus Ntg.

**Table 1:** Morphology data from female IGF1R Tg (physiological model) and DCM-dnPI3K Tg (pathological model)

	Physiological hypertrophy		Pathological hypertrophy	
	Ntg	IGF1R Tg	Ntg	DCM-dnPI3K Tg
**No. of animals**	10	7	8	7
**Age (weeks)**	21.7 ± 0.0	21.7 ± 0.0	20.4 ± 0.2	20.5 ± 0.1
**Body Weight (g)**	27.3 ± 0.8	27.0 ± 1.1	28.9 ± 0.8	31.6 ± 1.0*
**Tibia Length (TL, mm)**	16.8 ± 0.1	16.6 ± 0.1	16.8 ± 0.1	17.3 ± 0.1*
**Heart Weight (HW, mg)**	99.6 ± 2.0	134.5 ± 2.7**	101.9 ± 1.5	122.7 ± 3.6**
**Left Ventricle (LV) W (mg)**	70.3 ± 1.3	96.7 ± 2.4**	74.3 ± 1.4	81.2 ± 2.0*
**Right Ventricle (RV) W (mg)**	21.0 ± 0.7	26.6 ± 0.7**	20.5 ± 0.6	24.0 ± 0.7**
**Atria Weight (mg)**	6.4 ± 0.5	8.9 ± 0.3**	5.5 ± 0.4	14.7 ± 0.8**
**Left Atria Weight (mg)**	2.9 ± 0.3	4.8 ± 0.3**	2.4 ± 0.3	5.2 ± 0.3**
**Right Atria Weight (mg)**	3.4 ± 0.3	3.9 ± 0.2	3.2 ± 0.4	9.2 ± 0.4**
**Lung Weight (LW, mg)**	132.3 ± 5.0	129.4 ± 3.2	135.8 ± 4.9	187.0 ± 6.3**
**HW/TL (mg/mm)**	5.93 ± 0.10	8.13 ± 0.14**	6.05 ± 0.08	7.10 ± 0.18**
**LV/TL (mg/mm)**	4.18 ± 0.06	5.84 ± 0.14**	4.41 ± 0.08	4.70 ± 0.10*
**RV/TL (mg/mm)**	1.25 ± 0.04	1.61 ± 0.04**	1.22 ± 0.04	1.39 ± 0.04**
**LW/TL (mg/mm)**	7.88 ± 0.30	7.82 ± 0.17	8.07 ± 0.29	10.84 ± 0.38**

Abbreviations: DCM-dnPI3K Tg, Tg mice with dilated cardiomyopathy (DCM) and cardiac-specific transgenic expression of dominant negative phosphoinositide 3-kinase (dnPI3K); IGF1R Tg, insulin-like growth factor 1 receptor cardiac-specific transgenic mice; Ntg, non-transgenic mice (littermate controls); W, weight. Data are shown as mean ± SEM. *P<0.05 vs. Ntg littermate control, **P<0.01 vs. Ntg littermate control, using an unpaired *t*-test.

**Table 2:** Morphology data from male IGF1R Tg (physiological model) and DCM-dnPI3K Tg (pathological model)

	Physiological hypertrophy		Pathological hypertrophy	
	Ntg	IGF1R Tg	Ntg	DCM-dnPI3K Tg
**No. of animals**	8	9	6	7
**Age (weeks)**	19.5 ± 0.2	19.6 ±0.2	20.4 ± 0.4	20.0 ± 0.2
**Body Weight (g)**	35.2 ± 1.1	35.8 ± 0.9	39.1 ± 1.5	40.1 ± 0.8
**Tibia Length (TL, mm)**	16.8 ± 0.1	16.8 ± 0.1	17.2 ± 0.2	17.1 ± 0.1
**Heart Weight (HW, mg)**	130.2 ± 2.9	173.8 ± 3.4**	142.3 ± 3.9	128.4 ± 4.9
**Left Ventricle (LV) W (mg)**	93.7 ±1.9	123.8 ± 2.1**	105.0 ± 2.9	90.4 ± 3.6*
**Right Ventricle (RV) W (mg)**	24.3 ± 0.7	32.3 ± 1.1**	26.9 ± 1.1	24.0 ± 0.9
**Atria Weight (mg)**	9.4 ± 0.4	14.2 ± 0.6**	7.7 ± 0.3	12.6 ± 0.4**
**Left Atria Weight (mg)**	4.5 ± 0.2	7.7 ± 0.4**	3.8 ± 0.2	4.4 ± 0.3
**Right Atria Weight (mg)**	5.0 ±0.2	6.5 ± 0.3**	3.9 ± 0.2	8.2 ± 0.3**
**Lung Weight (LW, mg)**	142.7 ± 4.5	153.5 ± 3.9	147.3 ± 9.1	167.7 ± 12.7
**HW/TL (mg/mm)**	7.73 ± 0.16	10.33 ± 0.22**	8.30 ± 0.28	7.49 ± 0.27
**LV/TL (mg/mm)**	5.57 ± 0.10	7.35 ± 0.13**	6.12 ± 0.19	5.27 ± 0.20*
**RV/TL (mg/mm)**	1.44 ± 0.04	1.92 ± 0.06**	1.57 ± 0.07	1.40 ± 0.05
**LW/TL (mg/mm)**	8.47 ± 0.24	9.11 ± 0.22	8.59 ± 0.54	9.77 ± 0.71

Abbreviations: DCM-dnPI3K Tg, Tg mice with dilated cardiomyopathy (DCM) and cardiac-specific transgenic expression of dominant negative phosphoinositide 3-kinase (dnPI3K); IGF1R Tg, insulin-like growth factor 1 receptor cardiac-specific transgenic mice; Ntg, non-transgenic mice (littermate controls); W, weight. Data are shown as mean ± SEM. *P<0.05 vs. Ntg littermate control, **P<0.01 vs. Ntg littermate control, using an unpaired *t*-test.

**Figure 1:**
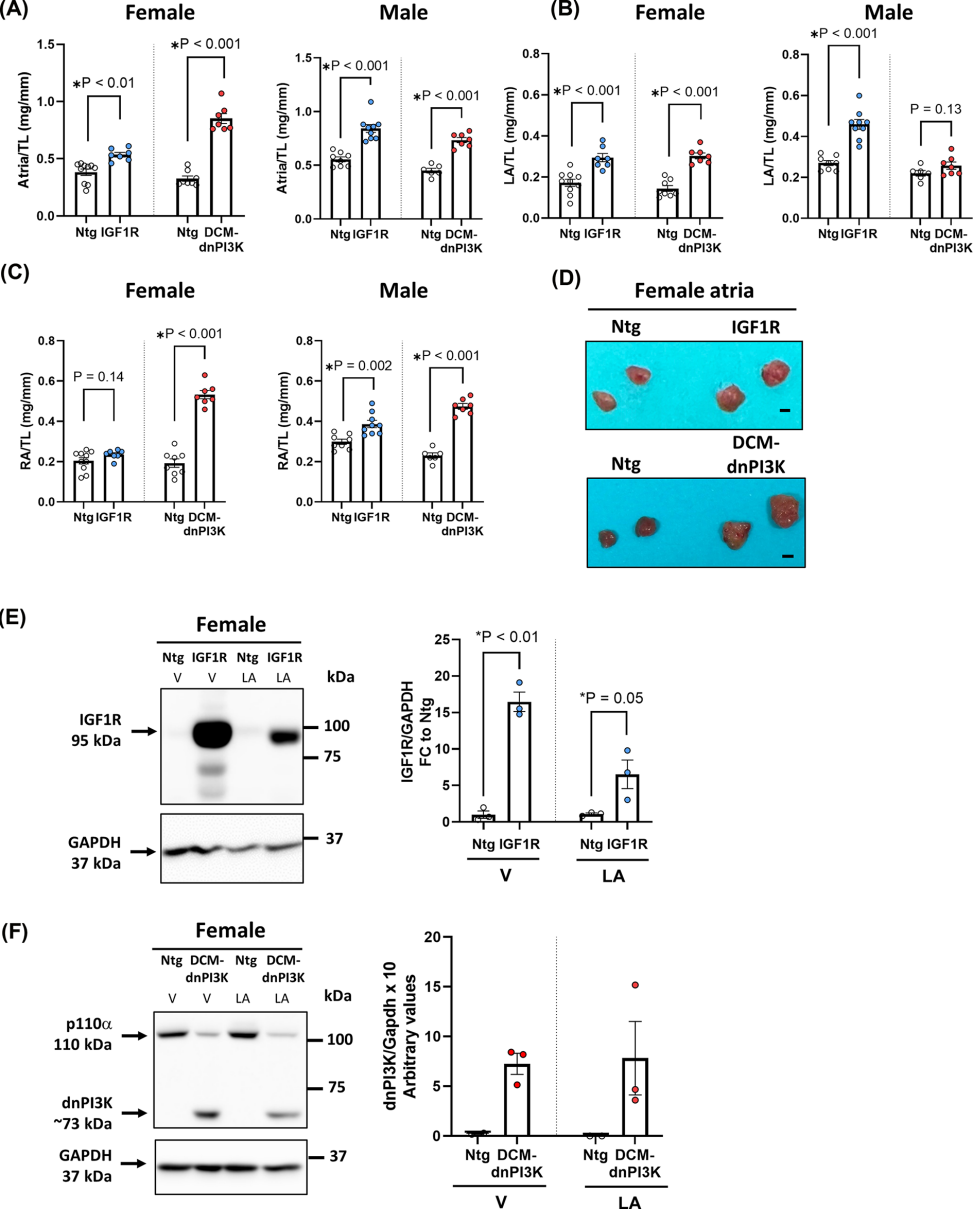
Atria characterisation in mouse models of physiological and pathological cardiac hypertrophy (**A)** Total atria weight, (**B**) left atrial (LA) weight and (**C**) right atrial (RA) weight normalised to tibia length (TL) in the physiological model (IGF1R Tg) and pathological model (DCM-dnPI3K Tg) at ∼20 weeks of age. Ntg = non-transgenic controls. Female mice: *n* = 7–10. Male mice: *n* = 6–9. Data are presented as mean ± SEM. All data passed the normality test. Groups compared using unpaired t-test. **(D)** Representative atria of female mice. Scale bar = 0.1 cm. **(E)** Representative Western blot and quantitation of IGF1R protein expression (normalised to GAPDH) in ventricles (V) and left LA of female IGF1R and Ntg mice. Unpaired *t*-tests (*n* = 3/group). (**F**) Representative Western blot and quantitation of dnPI3K protein expression in ventricles (V) and LA of female DCM-dnPI3K and Ntg mice (*n* = 3/group). N.B. Statistics are not presented on panel F because the dnPI3K band is an ‘all or none’ response given Ntg mice don’t endogenously express the mutant dnPI3K. The relative difference is in reference to the background signal/noise on the blot.

Total atria weight normalised to tibia length was increased in the physiological and pathological model versus Ntg littermate controls (Figure 1A), as previously reported [15,16]. In the physiological model, there was an increase in LA weight normalised to tibia length (LA/TL) in both sexes (∼70% increase for both sexes; Figure 1B). RA enlargement was less pronounced and only statistically significant in male IGF1R mice (Figure 1C). In the pathological model, significant LA enlargement was observed in female, but not male, DCM-dnPI3K mice versus Ntg control (Figure 1B), and RA enlargement was observed in both sexes (female: ∼177% increase and male: ∼106% increase; Figure 1C). As the increase in LA weight in female mice was similar between the physiological and pathological models (∼2-fold increase vs. Ntg littermate controls, Figure 1B,D), and the goal of the present study was to compare and contrast physiological and pathological atrial enlargement (independent of differences in atrial mass), the remainder of the study focused on LA from female mice.

To confirm activity of the αMHC promoter and its ability to drive transgene expression in atrial tissue (as previous investigations have largely studied transgene expression in the ventricle), we investigated transgene expression in atrial tissue via Western blot. Robust overexpression of the IGF1R was observed in ventricular and atrial tissue of IGF1R Tg mice (Figure 1E). The dnPI3K transgene was detected in DCM-dnPI3K Tg ventricles and atria, but not Ntg cardiac tissues (Figure 1F).

### Pathological, but not physiological atrial enlargement, is associated with atrial dysfunction

**Figure 2:**
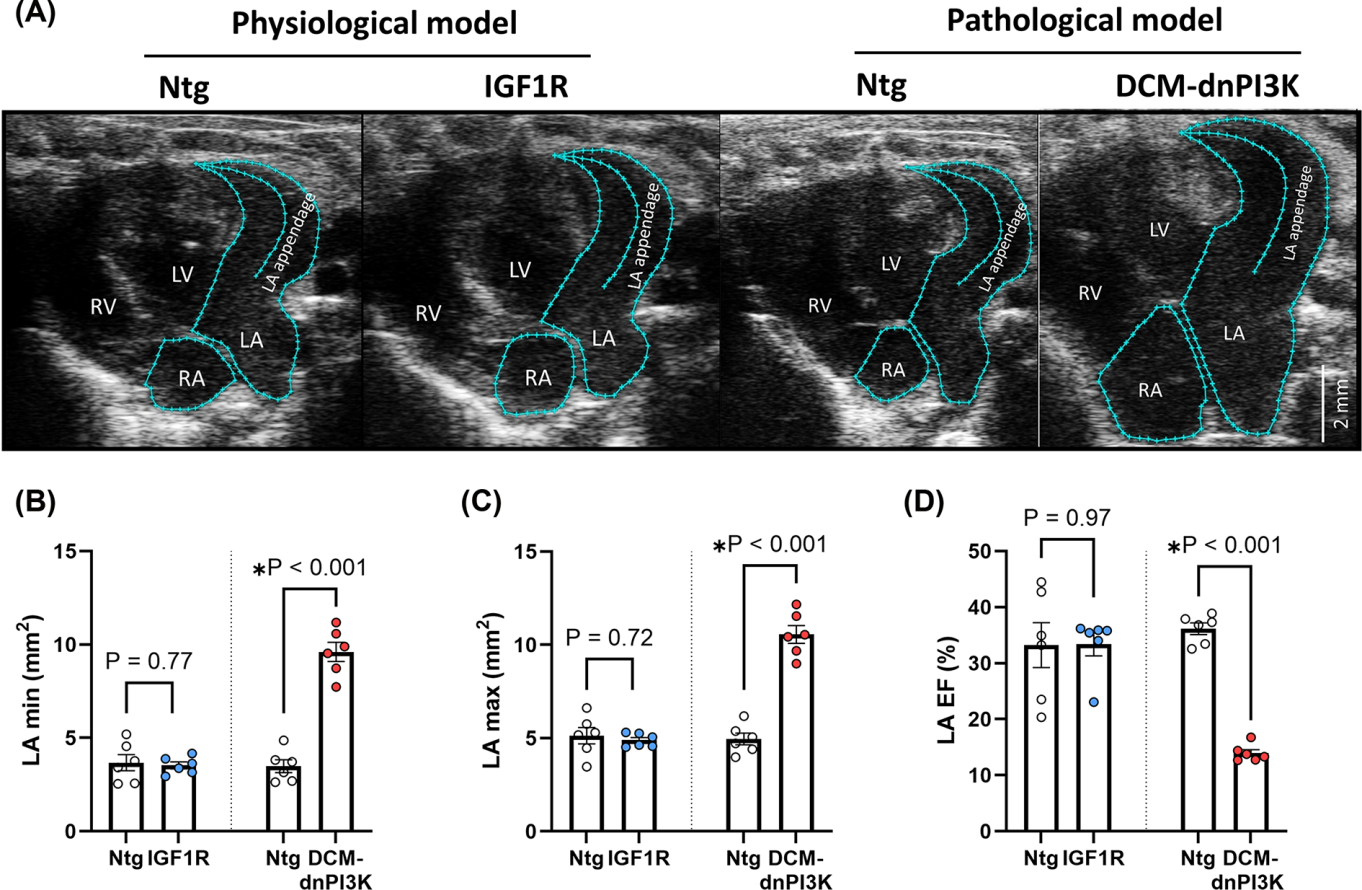
Echocardiographic assessment of left atrial dimensions and function in female mice with physiological or pathological cardiac hypertrophy (**A**) Representative echocardiograms from female mice in the physiological model (IGF1R Tg) and pathological model (DCM-dnPI3K Tg) versus Ntg at ∼20 weeks of age. (**B–D**) LA systolic area (LA min), LA diastolic area (LA max), LA ejection fraction (LA EF), *n* = 6/group. Data are presented as mean ± SEM. All data passed the normality test. Unpaired *t*-test.

To determine if there were differences in LA function between the IGF1R Tg and DCM-dnPI3K Tg, we performed transthoracic echocardiography on female mice at 20 weeks of age (Figure 2A). Despite the increase in LA weight in the IGF1R Tg versus Ntg, LA areas and volumes at systole (LA_min_, Figue 2B and Table 3) and diastole (LA_max_, Figure 2C and Table 3) were not significantly greater than Ntg. In contrast, LA areas and volumes were increased in DCM-dnPI3K mice versus Ntg littermates (∼3-fold increase in LA_min_, ∼2-fold increase in LA_max_; *P*<0.001; Figure 2B,C and Table 3). Atrial function, as determined by LA ejection fraction (LA EF) was preserved in IGF1R mice, but reduced in the DCM-dnPI3K mice (∼60% decrease in LA EF in DCM-dnPI3K vs. Ntg, *P*<0.001; Figure 2D). Additional measures of atrial dimensions and function of the LA, LA and appendage (LA+APP) and RA are provided in Table 3.

**Table 3:** Echocardiography data from female IGF1R Tg (physiological model) and DCM-dnPI3K Tg (pathological model)

	Physiological hypertrophy		Pathological hypertrophy	
	Ntg	IGF1R	Ntg	DCM-dnPI3K
**No. of animals**	6	6	6	6
**Age (weeks)**	21.4 ± 0.1	21.4 ± 0.1	19.9 ±0.2	20.0 ± 0.1
**Body Weight (g)**	27.5 ± 1.6	27.6 ± 1.2	29.8 ± 0.7	32.6 ± 1.0*
**Heart Rate (bpm)**	457 ± 13	455 ± 8	473 ± 16	495 ± 12
**LA Volume pre-A (μl)**	6.4 ± 0.8	6.2 ± 0.2	6.3 ± 0.8	20.1 ± 1.4**
**LA Volume min (μl)**	5.5 ± 0.9	4.9 ± 0.4	4.7 ± 0.5	18.6 ± 1.5**
**LA Volume max (μl)**	8.0 ± 0.9	7.3 ± 0.4	7.3 ± 0.7	21.6 ± 1.6**
**LA reservoir function**				
LA Total Emptying Volume (μl)	2.6 ± 0.3	2.4 ± 0.2	2.6 ± 0.2	3.0 ± 0.1
LA Total Emptying Fraction	0.33 ± 0.04	0.33 ± 0.02	0.36 ± 0.01	0.14 ±0.01**
**LA conduit function**				
LA Passive Emptying Volume (μl)	1.6 ± 0.4	1.0 ± 0.2	1.0 ± 0.2	1.5 ± 0.3
LA Passive Emptying Fraction	0.20 ± 0.05	0.14 ±0.03	0.15 ±0.04	0.07 ± 0.01 (P = 0.07)
**LA booster pump function**				
LA Active Emptying Volume (μl)	1.0 ± 0.2	1.4 ± 0.2	1.6 ± 0.4	1.5 ± 0.3
LA Active Emptying Fraction	0.16 ± 0.03	0.22 ± 0.04	0.24 ± 0.03	0.08 ± 0.02**
**LA+APP Area min (mm^2^)**	7.0 ± 1.0	7.3 ± 0.4	6.3 ± 0.6	18.8 ± 1.1**
**LA+APP Area max (mm^2^)**	9.9 ± 0.9	10.5 ± 0.4	9.7 ± 0.8	20.9 ± 1.0**
**LA+APP Total Emptying Fraction**	0.31 ± 0.03	0.31 ± 0.02	0.36 ± 0.02	0.10 ± 0.01**
**RA Area min (mm^2^)**	1.8 ± 0.2	2.0 ± 0.1	1.6 ± 0.2	7.3 ± 0.6**
**RA Area max (mm^2^)**	2.7 ± 0.2	3.0 ± 0.1	2.4 ± 0.3	8.0 ± 0.6**
**RA Total Emptying Fraction**	0.34 ± 0.02	0.33 ± 0.02	0.35 ± 0.01	0.10 ± 0.01**

Abbreviations: DCM-dnPI3K Tg, Tg mice with dilated cardiomyopathy (DCM) and cardiac-specific transgenic expression of dominant negative phosphoinositide 3-kinase (dnPI3K); bpm, beats per minute; LA, left atrium; LA+APP, left atrium plus atrial appendage; IGF1R Tg, insulin-like growth factor 1 receptor cardiac-specific transgenic mice; Ntg, non-transgenic mice (littermate controls); RA, right atrium. Data are shown as mean ± SEM. *P<0.05 vs. Ntg littermate control, **P<0.01 vs. Ntg littermate control, using an unpaired t-test.

### Atrial enlargement in settings of physiological and pathological cardiac remodelling is accompanied by differences in atrial myocyte morphology

**Figure 3:**
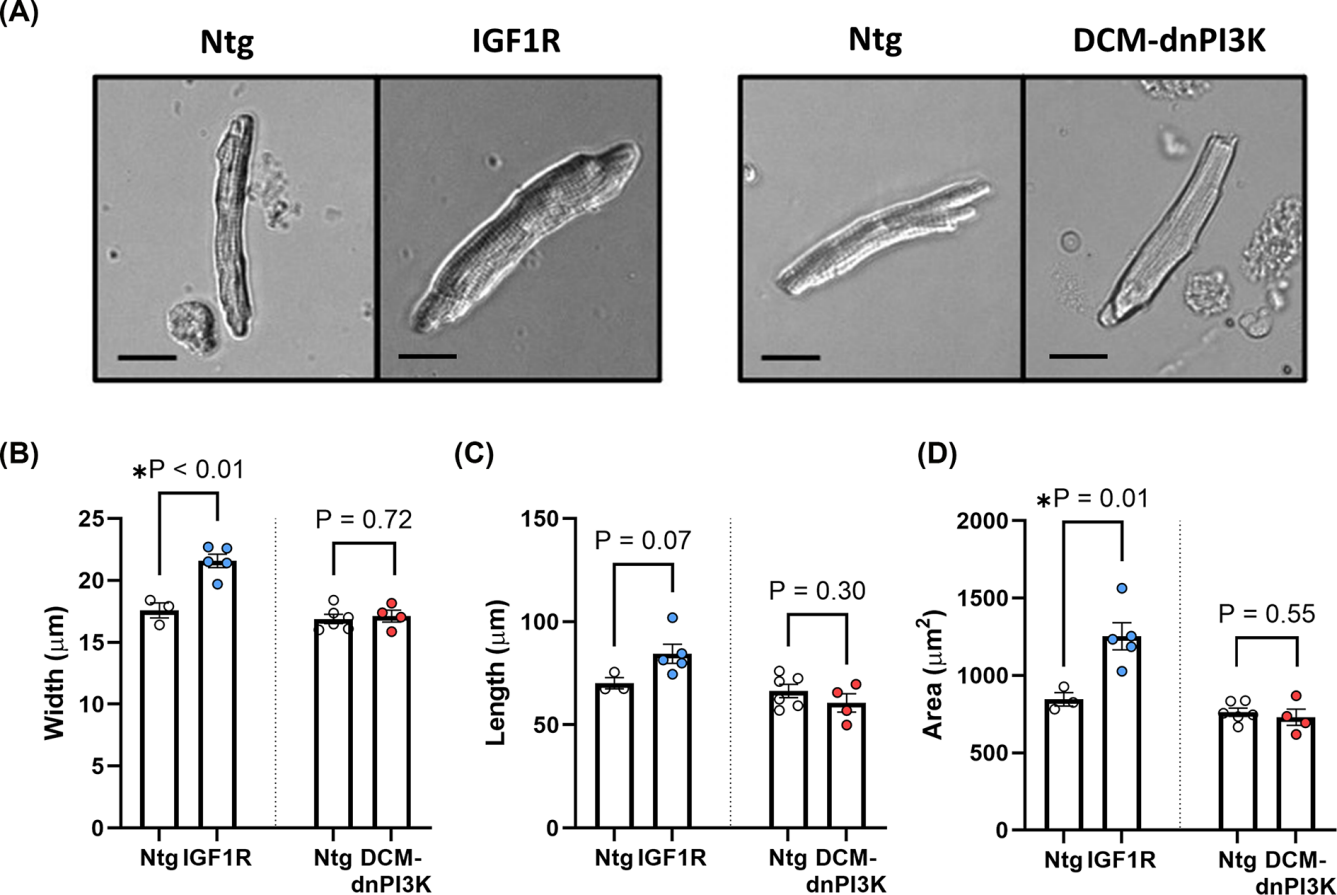
Atrial myocyte dimensions in female mice with physiological or pathological cardiac hypertrophy (**A**) Atrial myocytes isolated from hearts of mouse models of IGF1R Tg and DCM-dnPI3K Tg versus Ntg at ∼20 weeks of age. Atrial myocyte width (**B**), length (**C**) and area **(D**). IGF1R model: 40–170 myocytes were measured from 3 to 5 animals/group. DCM-dnPI3K model: 28–86 myocytes were measured from 4 to 6 animals/group. Data are mean ± SEM. Unpaired *t*-tests. Scale bar: 20 μm.

**Figure 4:**
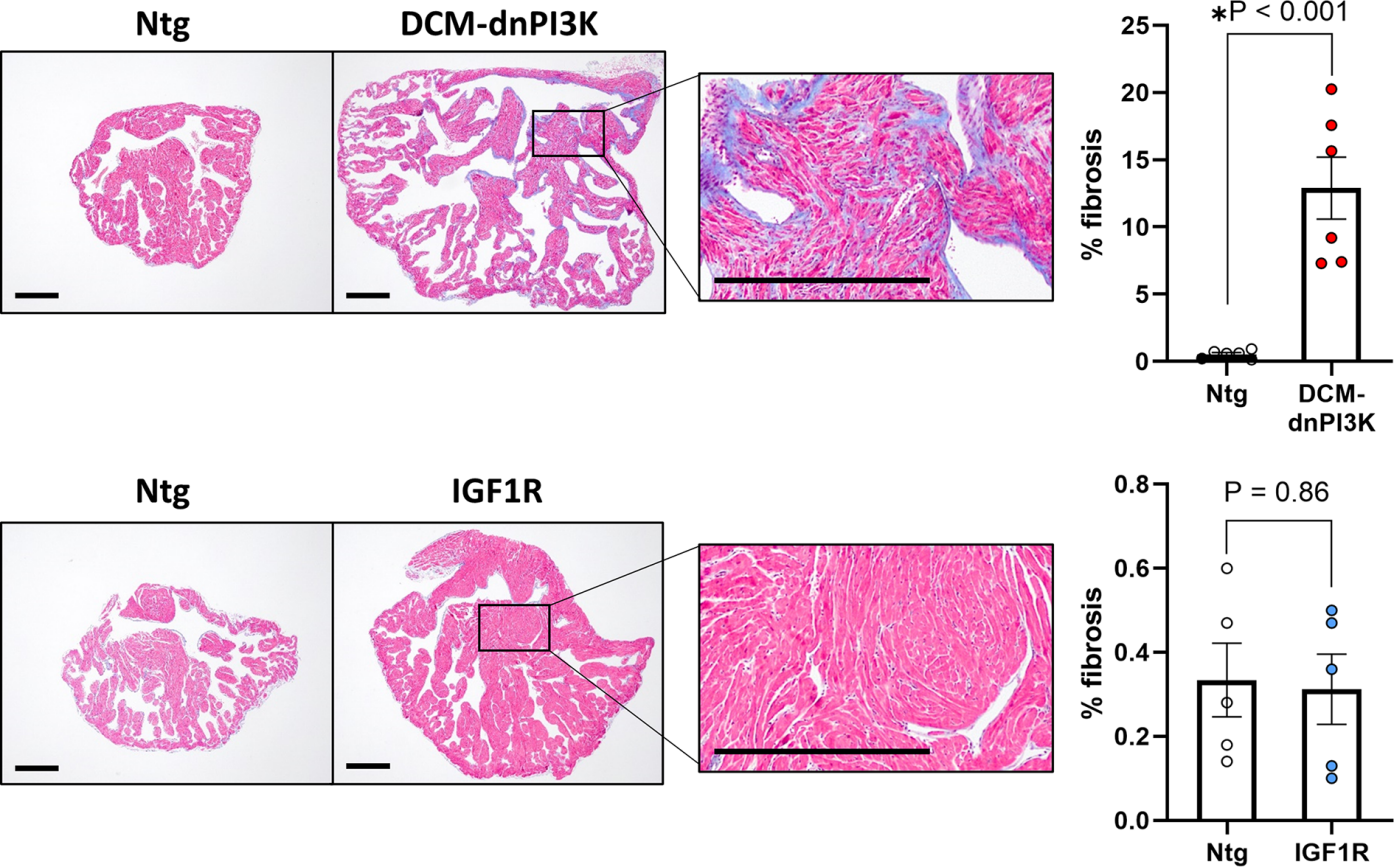
Histological assessment of left atrial fibrosis in female mice with physiological or pathological cardiac hypertrophy Histological examination of LA sections stained with Masson’s Trichrome and quantitation of LA fibrosis in 20 week-old female mouse models of physiological (IGF1R Tg) and pathological cardiac hypertrophy (DCM-dnPI3K Tg) vs. Ntg. Data are mean ± SEM. Unpaired *t*-tests. *n* = 5–6/group. Scale bar: 0.5 mm.

Next, we isolated myocytes from hearts of IGF1R Tg and DCM-dnPI3K Tg to determine if differences in atrial morphology and function were accompanied by differences in atrial myocyte dimensions. Consistent with the original characterisation [12], ventricular myocytes from IGF1R mice were wider (∼10% increase, *P*=0.04; Table 4) and tended to have a greater area than Ntg ventricular myocytes (∼11% increase, *P*=0.09; Table 4). Atrial myocytes from IGF1R mice were significantly wider than Ntg control (∼22% increase, *P*=0.003; Figure 3A,B), tended to be longer (∼20% increase; *P*=0.07; Figure 3C) and had larger areas (∼48% increase, *P*=0.01; Figure 3D), indicating the addition of sarcomeres in parallel and in series. In the pathological model, ventricular myocytes of DCM-dnPI3K model were shorter than Ntg, consistent with a prior report [22]. Interestingly, there were no differences in atrial myocyte width, length or area between Ntg and DCM-dnPI3K mice (Figure 3A−D), suggesting that differences in atrial weight (Figure 1) were due to an expansion of extracellular matrix or differences in the non-myocyte cell population within the atria rather than myocyte hypertrophy. Histological assessment of fibrosis in LA sections showed a significant increase in interstitial collagen deposition in DCM-dnPI3K Tg compared with Ntg controls (*P*<0.001, Figure 4). In contrast, no differences in LA collagen deposition were observed between the physiological model and Ntg controls (Figure 4). Histological assessment also highlighted more dense myocyte muscle area within atria of IGF1R Tg versus DCM-dnPI3K Tg; explaining the increased LA weight in IGF1R Tg in the absence of a significant increase in LA areas and volumes by echocardiography. At higher magnification, significant disruption of cardiomyocytes and infiltration of other cells types were visible in the LA of DCM-dnPI3K Tg in comparison with Ntg and the IGF1R model (Supplementary Figure S2).

**Table 4:** Dimensions of ventricular myocytes isolated from female IGF1R and DCM-dnPI3K mice and non-transgenic (Ntg) controls at 20 weeks of age

	Physiological model		Pathological model	
	Ntg (*n*=3)	IGF1R (*n*=4)	Ntg (*n*=5)	dnPI3K-Mst1 (*n*=4)
Body weight (g)	29.8 ± 0.7	28.0 ± 1.0	33.7 ± 1.3	33.6 ± 3.1
Length (μm)	132 ± 5	131 ± 4	137 ± 3	109 ± 3 * (*P*=0.0002)
Width (μm)	32 ± 1	35 ± 0 * (*P*=0.04)	30 ± 1	32 ± 1
Area (μm^2^)	2934 ± 171	3250 ± 41 (*P*=0.09)	2985 ± 176	2679 ± 210

Physiological model: *n* = 76–123 ventricular myocytes from 3 to 4 animals per group. Pathological model: *n* = 74–93 ventricular myocytes from 4 to 5 animals per group. Data are mean ± SEM of the mean length/width/area from each animal. *P<0.05, unpaired *t*-test vs. Ntg.

### Differential gene expression in physiological and pathological atria

**Figure 5:**
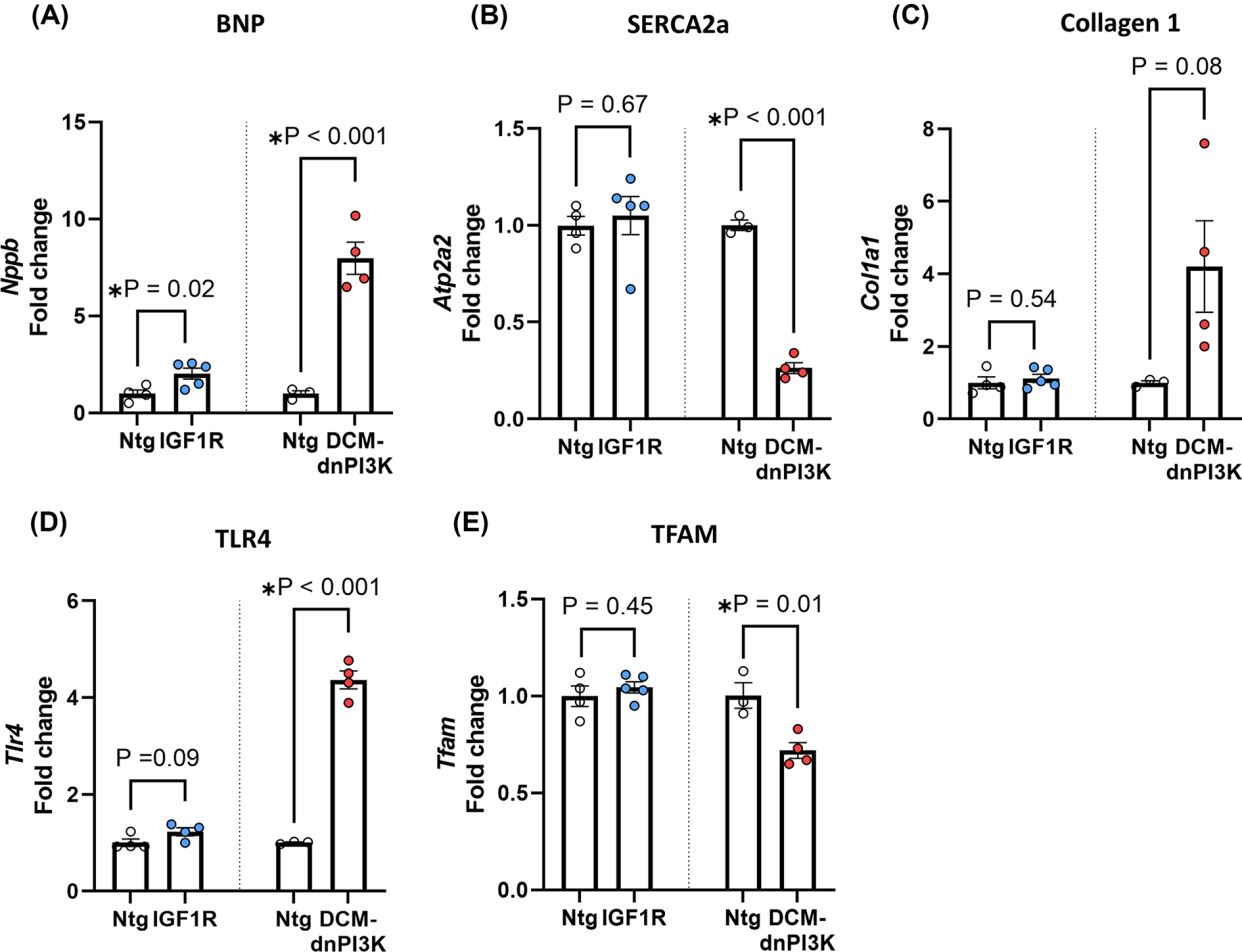
Left atrial gene expression in female mice with physiological or pathological cardiac hypertrophy qPCR assessment of gene expression in female mouse models of physiological (IGF1R Tg) and pathological cardiac hypertrophy (DCM-dnPI3K Tg) vs. Ntg at ∼20 weeks of age. (**A**) B-type natriuretic peptide (BNP, *Nppb*), (**B**) sarcoplasmic/endoplasmic reticulum calcium-ATPase 2a (SERCA2a, *Atp2a2*) Serca2a, (**C**) collagen 1 (*Col1a1*), (**D**) toll-like receptor 4 (*Tlr4*), and (**E**) transcription factor A, mitochondrial (TFAM). Unpaired *t*-test (A, C–E). For data that failed the normality test (TLR4 gene expression Ntg vs. IGF1R, (D)), a Mann–Whitney test was performed.

Physiological and pathological ventricular hypertrophy are associated with distinct gene expression signatures [34]. To determine whether similar differences occur in the atria, we performed qPCR to quantify the expression of key genes that are typically altered in settings of pathological, but not physiological, cardiac hypertrophy. Expression of B-type natriuretic peptide (BNP, encoded by *Nppb*) was modestly elevated in the LA of the IGF1R model (∼2-fold increase) as previously reported in the ventricle [35], but was elevated to a much greater degree in the DCM-dnPI3K model (∼8-fold increase; Figure 5A). Expression of the sarcoplasmic reticulum calcium pump (SERCA2a, encoded by *Atp2a2*) was similarly expressed in IGF1R and Ntg atria, but expression significantly reduced in atria of DCM-dnPI3K mice compared with Ntg controls (Figure 5B). The fibrosis marker collagen I (encoded by *Col1a1*) and the inflammatory marker toll-like receptor 4 (TLR4, encoded by *Tlr4*) were both upregulated in DCM-dnPI3K atria versus Ntg, but were not different in IGF1R atria (Figure 5C,D). Finally, we observed a significant reduction in the expression of transcription factor A, mitochondrial (TFAM, encoded by *Tfam*) in DCM-dnPI3K atria versus Ntg, but not in IGF1R atria (Figure 5E). Collectively, this highlights a distinct gene expression profile between the atria from the physiological and pathological models.

### Proteomic profiling of atrial tissues in pathological versus physiological cardiac hypertrophy models

To characterise differences in protein expression and signalling networks in atria of IGF1R and DCM-dnPI3K mice, we performed proteomics on LA tissue from the two models (physiological and pathological, with matched Ntg controls; *n* = 3/group). Proteomic analysis was performed in LA from mice at 8 weeks of age because we wanted to select a time point at which postnatal growth is complete, while still capturing relative early changes in protein expression, i.e. before more significant established pathology in the DCM-dnPI3K Tg model. At 8 weeks of age, atrial enlargement was evident in both IGF1R Tg and DCM-dnPI3K Tg versus Ntg (Supplementary Figure S3A). In the DCM-dnPI3K model, there was also evidence of atrial dysfunction and gene expression changes at 8 weeks versus Ntg, but the increase in BNP was smaller (∼4-fold at 8 weeks vs. 8-fold at 20 weeks) and there was no significant fall in TFAM (Supplementary Figure S3B).

**Figure 6:**
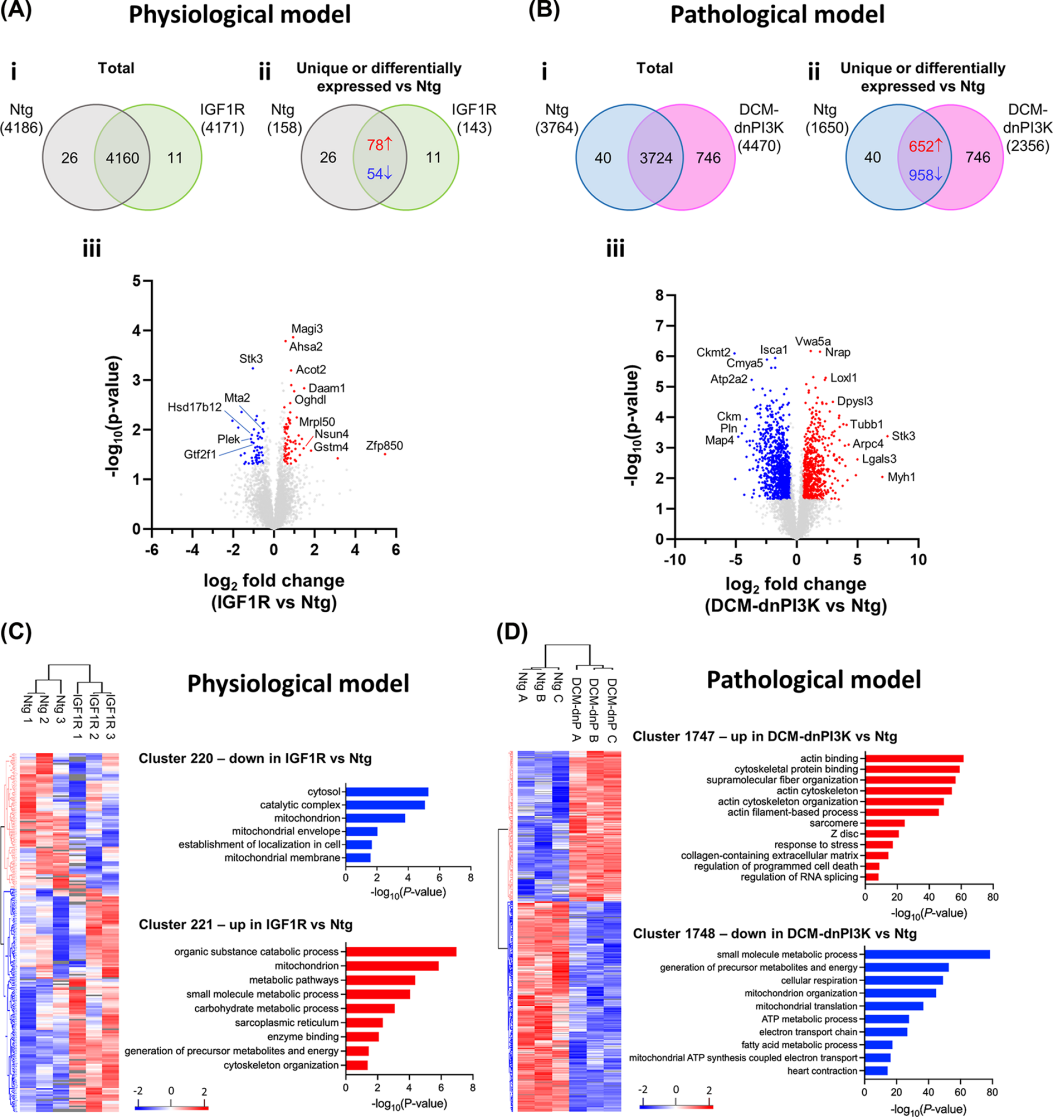
Proteomic characterisation of left atria from mouse models of physiological and pathological cardiac hypertrophy Quantitative proteomic profiling was performed on proteins extracted from female LA tissue of mouse models of physiological (IGF1R Tg) and pathological cardiac hypertrophy (DCM-dnPI3K Tg) versus Ntg at 8 weeks of age (**A**) (i) Venn diagram depicting total number of proteins quantified in all three biological replicates for each group. (ii) Venn diagram depicting number of proteins uniquely identified within each group (all replicates) and differentially expressed abundant proteins in IGF1R vs Ntg (±0.5 log_2_ fold change, *P*<0.05). (iii) Volcano plot with dysregulated protein abundance (±0.5 log_2_ fold change) in IGF1R versus Ntg, *P*<0.05 by unpaired *t*-test. (**B**) (i) Venn diagram depicting total number of proteins quantified in all three biological replicates for each group. (ii) Venn diagram depicting number of proteins uniquely identified within each group and differentially abundant proteins in DCM-dnPI3K versus Ntg. (iii) Volcano plot with dysregulated protein abundance (±0.5 log_2_ fold change) in DCM-dnPI3K versus Ntg, *P*<0.05 by unpaired *t*-test. (**C,D**) Clustered protein expression heatmaps for the physiological and pathological models (*P*<0.05 by unpaired *t*-test; *z*-score normalised). Significant biological processes, molecular functions and cellular components (Gene Ontology Enrichment Analysis using gProfiler, term size 5-5000) associated with each cluster shown (blue, down-regulation; red, up-regulation).

Principal component analysis of the proteomic data showed the greatest difference between the DCM-dnPI3K model versus Ntg (Supplementary Figure S4). A total of 4197 and 4510 proteins were quantified in the physiological and pathological models, respectively (Figure 6Ai, Bi; Supplementary Tables 3.1 and 3.2). With stringent inclusion criteria, in the physiological model we observed increased abundance of 78 proteins and reduced abundance of 54 proteins in IGF1R versus Ntg atria (Figure 6Aii, iii; Supplementary Tables 3.3 and 3.3i). A greater number of proteins were differentially abundant in the atria of DCM-dnPI3K versus Ntg mice (652 up-regulated, 958 down-regulated; Figure 6Bii, iii; Supplementary Tables 3.4 and 3.4i), which was not unexpected given the more pronounced functional and histological phenotype in the pathological model. Amongst the significantly differentially abundant proteins in IGF1R atria were zinc finger protein 850 (Zfp850, ∼5.5 log_2_ fold increase), glutathione S-transferase mu 4 (Gstm4, ∼1.8 log_2_ fold increase), membrane associated guanylate kinase, WW and PDZ domain containing 3 (Magi3, ∼0.9 log_2_ fold increase), and serine/threonine kinase 3 (Stk3, ∼1 log_2_ fold decrease). In DCM-dnPI3K atria, Stk3 (∼7.5 log_2_ fold increase), myosin 1 (Myh1, ∼7 log_2_ fold increase) and galectin-3 (Lgals3, ∼5 log_2_ fold increase) were the most significantly up-regulated proteins. Mitochondrial creatine kinase 2 (Ckmt2), SERCA2a (Atp2a2), microtubule-associated protein (Map4) and phospholamban (Pln) were amongst the most significantly down-regulated proteins in the pathological model (∼4.5–5.1 log_2_ fold decrease).

Gene Ontology enrichment analysis revealed protein networks down-regulated (significantly [*P*<0.05] differentially expressed) in IGF1R atria versus Ntg (cluster 220) that were mostly associated with the mitochondria (Figure 6C, Supplementary Table S3.9), while biological processes upregulated in IGF1R atria (cluster 221) were associated with catabolism (e.g. organic substance catabolic process, Figure 6C, Supplementary Table S3.10) and metabolism (e.g. small molecule, carbohydrate, carboxylic acid, oxoacid and organic acid metabolic processes; generation of precursor metabolites and energy; Figure 6C, Supplementary Table S3.10). Collectively, these processes indicate metabolic remodelling of IGF1R atria. In the pathological model, there was an enrichment of terms associated with numerous biological processes (see Supplementary Tables 3.13 and 3.14). Terms associated with cluster 1748 (down-regulated proteins) related to mitochondria and energy production (e.g. cellular respiration, ATP metabolic process, electron transport chain, fatty acid metabolic process; Figure 6D, Supplementary Table S3.14) and cardiac function (e.g. heart contraction, Figure 6D, Supplementary Table S3.14) while terms associated with cluster 1747 (up-regulated proteins) related to structural remodelling of cardiomyocytes (e.g. supramolecular fiber organisation, actin cytoskeleton organisation, sarcomere, Z disc; Figure 6D, Supplementary Table S3.13) and the ECM (collagen-containing extracellular matrix).

Next, we performed a comparative analysis of proteins that were differentially expressed in atrial tissue in the pathological mouse model with atrial tissue from patients with AF [31,32] (Supplementary Table S3.4, columns O & P). Of the two main clusters of proteins that were up-regulated (cluster 1747) or down-regulated (cluster 1748) in the DCM-dnPI3K atria versus Ntg, 61 were also identified in atrial tissue from patients with human AF. Thirty-two co-identified proteins in cluster 1747 were associated with processes such as extracellular matrix, cytoskeleton organisation, organelle organisation, cell adhesion molecule binding and muscle cell differentiation. This included actin cytoskeletal proteins (LCP1, ARPC4, CORO1A), and calcium signalling proteins (CAPG). The 29 proteins co-identified in cluster 1748 were associated with collagen-containing extracellular matrix, mitochondrion, mitochondrial membrane and focal adhesion. It also included proteins associated with transcriptional regulation (FHL2, CLUH, MECP2), ion transporter (SLC25A3), and enzymatic activity (MAOB, FN3KRP, ALAD).

**Figure 7:**
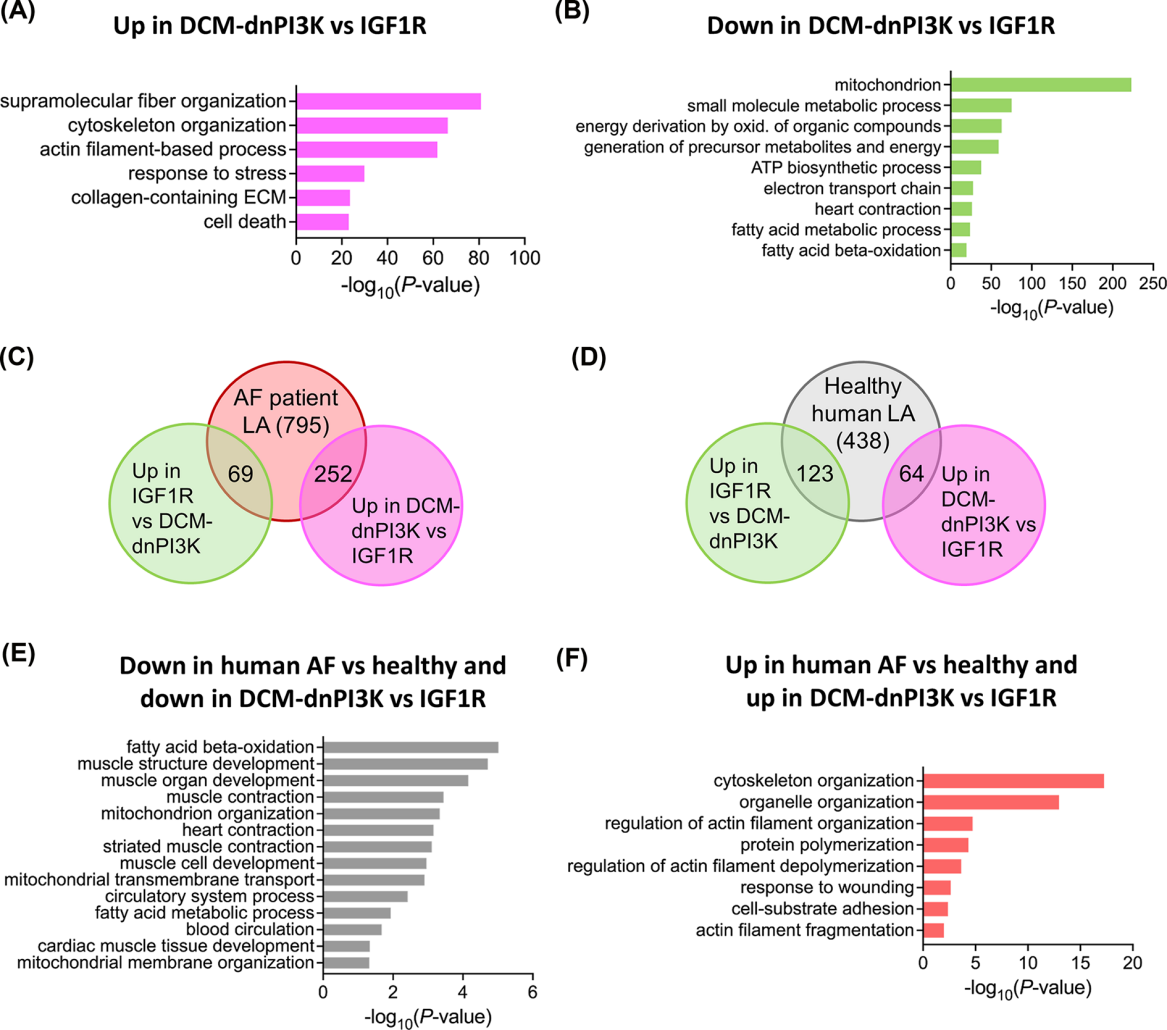
Differential mouse proteome analysis and comparative analysis with human atrial proteome Gene ontology enrichment of significant proteins up- (**A**) or down-regulated (**B**) in DCM-dnPI3K vs IGF1R (*P*<0.05, FC +/- 0.5). Biological processes, molecular functions and cellular components (Gene Ontology Enrichment Analysis using gProfiler, term size 5-5000). (**C, D**) Comparative analysis between mouse models and human data set (Doll et al.) [33]. Hierarchical clustering of LA from AF and healthy group revealed differentially expressed proteins in AF patients (795 proteins up-regulated in AF) compared with healthy human LA (438 proteins in healthy) based on ANOVA, FDR < 0.05. Based on similarity in co-identified proteins, the DCM-dnPI3K model comprises a greater proportion of proteins to human AF LA (252) than healthy human LA (69) (C), and the IGF1R model comprises a greater proportion of proteins to healthy LA (123) than AF LA (64) (D). N.B. UP in IGF1R vs DCM-dnPI3K is equivalent to DOWN in DCM-dnPI3K versus IGF1R (Supplementary Table S3.15). UP, *P*<0.05 (+0.5). (**E, F**) Gene ontology enrichment related to comparative differential proteome analysis of AF from human tissue with mouse models (Supplementary Table S3.15). (E) DOWN, *P*<0.05 (-0.5); (Supplementary Table S15, column P). (F**)** UP, *P*<0.05 (+0.5); (Supplementary Table S15, column Q).

To gain further understanding of the molecular mechanisms underlying the IGF1R and DCM-dnPI3K models, a direct comparison was performed together with enrichment analysis (Figure 7 and Supplementary Table S3.15). Gene Ontology enrichment analysis revealed protein networks upregulated in DCM-dnPI3K versus IGF1R related to fiber organisation, ECM and cell death (Figure 7A), and protein networks down-regulated that were associated with the mitochondria, metabolism and heart contraction (Figure 7B, Supplementary Table S3.15).

To further assess relevance to human AF, we also performed comparative differential proteome analysis including both human healthy atrial tissue and human AF atrial tissue [33] i.e. relative to DCM-dnPI3K versus IGF1R (Supplementary Table S3.15, columns P&Q). Based on similarity in co-identified proteins, the DCM-dnPI3K model is more similar to human AF LA (252 proteins) than healthy human LA (69 proteins; Figure 7C), and the IGF1R model is more similar to healthy LA (123 proteins) than AF LA (64 proteins; Figure 7D). Comparative analysis revealed 190/439 proteins co-identified in human AF (lowly expressed relative to healthy tissue), of which 125/190 were significantly down-regulated in expression in DCM-dnPI3K (associated with hallmark cardiac biological processes: fatty acid metabolism, heart/muscle contraction, mitochondria organisation/ function including MYH6, TPM1 and ACADM; Figure 7E, Supplementary Table S3.15). In contrast, 343/794 proteins were associated with human AF and significantly differentially expressed in DCM-dnPI3K versus IGF1R. These included 266/343 proteins up-regulated in expression associated with cytoskeletal and organelle regulation factors, including TPM4 and MYH10 (Figure 7F, Supplementary Table S3.15).

## Discussion

In comparison to the ventricle, functional and molecular characterisation of pathological and physiological atrial enlargement in mice has been very limited. This characterisation is important because pathological atrial enlargement typically progresses to AF and other atrial arrhythmias, whereas physiological atrial enlargement does not [8]. In the present study, we conducted a comprehensive investigation of atrial morphology and function in two distinct cardiomyocyte-specific transgenic mouse models with atrial enlargement. The first was a model of physiological cardiac hypertrophy driven by overexpression of the IGF1R (IGF1R Tg mice). The second was a model of pathological cardiac remodelling with DCM and increased susceptibility to AF due to reduced cardiac PI3K activity (DCM-dnPI3K Tg mice). Our study demonstrates that an increase in atrial mass in physiological and pathological settings differs at the functional, morphological, histological and molecular level. An understanding of the distinct mechanisms contributing to atrial remodelling and dysfunction in pathological settings versus the adaptive/protective remodelling in physiological settings has the potential to identify novel therapeutic strategies for the treatment of atrial myopathies and arrhythmias including AF.

Echocardiography is frequently used in experimental and preclinical models of heart disease to examine ventricular remodelling and function. Our study is one of only a handful of mouse studies to investigate atrial function by echocardiography. In our characterisation of LA from female IGF1R Tg and DCM-dnPI3K Tg mice, we found that there were differences in LA volumes and function, despite comparable LA weight. Of note, LA systolic and diastolic volumes were not increased and atrial function was preserved in the IGF1R model, while LA volumes were significantly increased and function was impaired in the DCM-dnPI3K model. LA dilation in the DCM-dnPI3K model was accompanied by significant fibrosis, which may arise from the apoptosis of atrial myocytes. Indeed, there was no difference in myocyte size in DCM-dnPI3K atria compared with Ntg, ruling out eccentric hypertrophy as the basis for LA dilation. In the IGF1R model, increased atrial myocyte size due to the addition of sarcomeres in parallel (increasing myocyte width) and in series (increasing myocyte length) contributed to an increase in atrial weight without an overall effect on internal chamber volume. These findings demonstrate that atrial enlargement can occur via different mechanisms (e.g. hypertrophy in physiological settings vs fibrosis and dilation in pathological settings), and that an increase in atrial mass does not necessarily correlate with a decline in atrial function.

Prior research has shown that exercise training or enhanced PI3K activity protect against *ventricular* dysfunction in mouse models of hypertrophic, dilated, and diabetic cardiomyopathy [17,20,36–38]. Conversely, reduced PI3K activity accelerates ventricular dysfunction and mortality in mice with DCM [15,17] and increases susceptibility to AF [15]. DCM-dnPI3K mice develop a pronounced HF phenotype, characterised by ventricular wall thinning, systolic dysfunction, atrial enlargement and fibrosis in the ventricles and atria [15]. In the current study, we show that this is accompanied by significant impairments in atrial function (i.e. reduced LA ejection fraction) and extensive remodelling of the atrial proteome, in the absence of atrial myocyte hypertrophy. Collectively, these findings support a protective role for PI3K signalling in both the ventricles and the atria.

Our gene expression studies revealed that atrial enlargement in the pathological model was accompanied by upregulation of BNP and fibrosis marker collagen I, along with downregulation of SERCA2a and transcription factor A, mitochondrial (TFAM). In contrast, the physiological model displayed only modest changes in BNP expression, while SERCA2a, TFAM, and collagen I were largely unaffected; underscoring the presence of distinct molecular signatures in these models. Regular aerobic exercise induces atrial enlargement that is characteristic of physiological atrial remodelling which is reversible [8]. Rats subjected to prolonged balanced exercise training developed atrial hypertrophy without electrical, inflammatory, or fibrotic remodelling [9]. By contrast, extreme endurance exercise in humans and mice can lead to pathological atrial enlargement [8]. In mice, extreme exercise-induced atrial enlargement was accompanied by fibrosis, inflammation, tumor necrosis factor (TNF) α signalling activation, and increased arrhythmia susceptibility [39]. These pathological atrial changes persisted after exercise cessation and could be prevented but not reversed by TNFα or p38 inhibition [39]. In another study, soluble TNFα was found to drive the adverse atrial remodelling induced by extreme exercise [40], and ablation of TNF had selective effects in atria versus ventricles, with atrial cardiomyocyte-derived TNF being critical for negative exercise-mediated atrial remodelling and heightened AF risk [41]. Consistent with our study showing that reduced PI3K contributes to atrial pathology, IGF1 gene expression was lower in the atria of mice after 2 weeks of intense swim exercise; these mice develop atrial fibrosis and have an increased susceptibility to AF [42]. Together with our findings, these studies demonstrate that atrial remodelling is associated with different functional outcomes in different physiological contexts, likely as a result of activation or inactivation of distinct signalling pathways.

**Figure 8:**
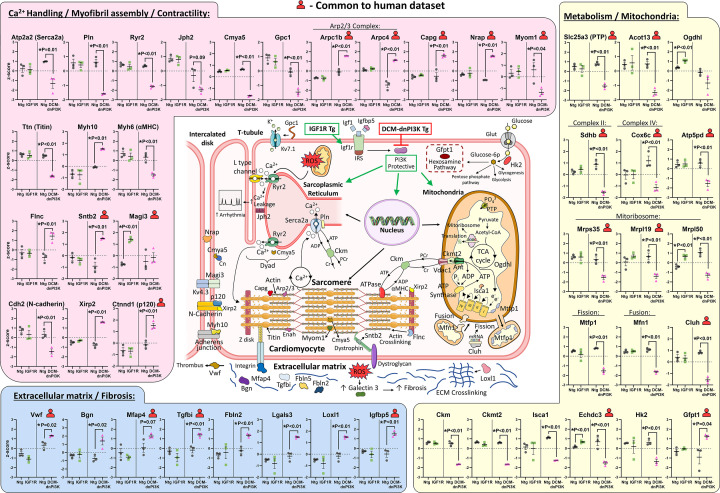
Overview of proteins differentially regulated in atria from the pathological and physiological model, and co-identification in human AF Regulation of proteins in LA from the physiological (IGF1R) and pathological model (DCM-dnPI3K). Proteins have been grouped into three main categories which are critical for atrial function: (1) Ca^2+^ handling/Myofibril assembly/Contractility (highlighted with pink shading), (2) Metabolism/Mitochondria (yellow shading), (3) Extracellular matrix/Fibrosis (blue shading). The majority of proteins were dysregulated in the pathological model but not the physiological model. Data points represent individual mice, presented as mean ±SEM. *N* = 3/group (normalised intensity,* z*-score). Unpaired *t*-test. Upper red human torso highlights those proteins also shown to be dysregulated in atrial tissue from patients with human AF. Protein names are defined in Supplementary Table S3.

To investigate molecular pathways contributing to the atrial phenotype of IGF1R and DCM-dnPI3K Tg mice in more detail, we performed proteomic analysis on LA tissue from female mice. Both models were associated with remodelling of the atrial proteome, with a greater number of proteins affected in the DCM-dnPI3K model compared with the IGF1R model (Figure 6). Interestingly, differential enrichment analyses revealed up-regulation of metabolic processes involving small molecules and carbohydrates in the physiological model. An increase in cardiomyocyte size (leading to an overall increase in heart size) increases the energy requirements of the heart. An increase in catabolic processes to produce energy is not unexpected in the IGF1R transgenic heart, and may explain why ventricular and atrial function is able to be maintained in this model. In contrast, in the DCM-dnPI3K there was a significant down-regulation of enrichment terms related to the production of energy. Down-regulation of proteins involved in energy production may mean the DCM-dnPI3K Tg heart is unable to meet its energy demands, leading to functional decline and contributing to heart failure pathogenesis [43]. These findings are consistent with those observed in atrial samples from patients with AF, in which we found common proteins between human AF and the DCM-dnPI3K model indicative of dysregulation of metabolism, mitochondria, and extracellular matrix. A cell surface proteoglycan, GPC1, was co-identified across the two proteomic studies on atrial tissue from human AF [31,32], and GPC1 was connected with voltage-gated K^+^ channels and ECG abnormalities in mice [44]. A summary of dysregulated proteins related to calcium handling/myofibril assembly/contractility, metabolic processes/mitochondria, and cardiomyocyte structural integrity/extracellular matrix/fibrosis are presented in Figure 8. In comparison to ventricular muscle, atrial muscle contracts faster (higher expression of αMHC and Serca2a) and contains a greater density of mitochondria that intercalate between myofilaments [21]. Thus, any dysregulation of proteins related to the contractile and metabolic apparatus are likely to contribute to atrial dysfunction and pathology (Figure 8).

Galectin-3 (Lgals3) is one example of a protein that was up-regulated in the DCM-dnPI3K atria (Figure 6Biii), but not the IGF1R atria (Figure 8), and has functional significance. We previously showed that deletion of galectin-3 attenuated LA enlargement (as determined by an increase in LA/TL ratio) and ECG abnormalities (i.e. prolonged P wave duration) in mice with DCM caused by overexpression of Mst1 [45]. Amongst other proteins that were significantly dysregulated in the DCM-dnPI3K model, a number were related to myofibril assembly, sarcomeres, contractility, and the intercalated disc (mechanical stress sensor). Cardiomyopathy-associated protein 5 (Cmya5, also known as myospryn; see Supplementary Table S3.4) was substantially down-regulated. CMYA5 is a large protein (∼450 kDa) with a number of locations and potential roles in cardiomyocytes [46–48] (Figure 8). Most recently, it was shown to be critical for maintaining the architecture and positioning of dyads, intracellular microdomains that bring Ca^2+^ channels in T-tubules and the junctional sarcoplasmic reticulum in close proximity for efficient Ca^2+^-induced Ca^2+^ release during excitation-contraction coupling [49]. Hearts lacking CMYA5 had reduced systolic function and LV dilation [49], similar to what is observed in DCM-dnPI3K mice. In the same study, CMYA5 was shown to play an important role in atrial cardiomyocytes (which lack T-tubules), tethering corbular sarcoplasmic reticulum containing RyR2 calcium channels to Z-lines [49]. Thus, mislocalisation of RyR2 due to down-regulation of CMYA5 may be a mechanism contributing to atrial dysfunction in the DCM-dnPI3K model. In the current study, Myh10 (nonmuscle myosin II-B) was elevated in the atria of DCM-dnPI3K Tg and Myh6 (αMHC) was depressed versus Ntg; no differences identified in the IGF1R Tg model. During cardiac development, switching between splicing isoforms of myofibril genes (e.g. myosin and titin) and Z-disk genes (e.g. ENAH, FLNC) is considered critical for normal sarcomere formation [46]. Initially, nonmuscle myosin II B (encoded by MYH10) integrates actin filament to form stress fiber-like structures, which are then organised into premyofibrils. Subsequently, premyofibrils assemble into developing/nascent myofibrils, and non-muscle myosin (MYH10) is replaced by muscle myosin (MYH6, αMHC) [50]. Sarcomere assembly was impaired in human embryonic stem cells during cardiac differentiation because MYH6 was unable to replace MYH10; leading to impaired myofibrillogenesis [46]. MYH10 was significantly reduced in mouse cardiomyocytes within one week after birth, but still detected in the intercalated disc of adult mice [51]. In the current study, a number of proteins related to the intercalated disc and actinin-2 binding proteins associated with DCM [46] were lower in the DCM-dnPI3K model (Figure 8 and Supplementary Figure S5).

### Clinical implications and future directions

The distinct functional and molecular phenotypes of the atria in physiological and pathological cardiac remodelling models provides an opportunity to identify new drug targets, but also has clinical implications for current drugs in use or in development for cancer. Many of the same pathways that protect the heart (e.g. PI3K) are inhibited by cancer therapies to halt and regress tumour growth [52,53]. The field of cardio-oncology has grown exponentially with a number of cancer agents leading to cardiotoxicity, including AF [54–56]. Given the multitude of biological processes that were affected in DCM-dnPI3K atria, therapeutic strategies to prevent atrial dysfunction and the associated risk of AF are likely to require a multi-pronged approach. Further studies are needed to evaluate the functional roles of specific proteins/signalling pathways underlying atrial dysfunction in pathological remodelling, and to examine therapeutic interventions. In the current study we focused on female mice because of the similarity in LA weight between female IGF1R Tg and female DCM-dnPI3K Tg mice. However, it would be of interest to study sex-specific differences in LA remodelling, as well as the RA in future work.

## Limitations

The current study evaluated myocyte size and collagen deposition within the atria. It would be of interest to study non-myocyte populations in future work, including the contribution of inflammation. Despite using an FDR of 1% for proteomics analysis, and focusing on proteins that were differentially regulated in the atria of the IGF1R Tg and DCM-dnPI3K Tg models, this does not completely eliminate the inclusion of proteins that may not be biologically significant. Verifying the contribution of proteins with unknown roles will be essential in future work. We selected the transgenic models based on characteristics, features and underlying mechanisms common to human AF and physiological conditions, i.e. a fall in IGF1R/PI3K signalling in human AF [15,16] and increased IGF1 with physiological hypertrophy in humans [57]. However, it is important to acknowledge that no transgenic mouse model can completely represent human AF and the physiological state.

## Conclusion

In summary, here we show that atrial enlargement associated with a model of physiological ventricular remodelling and pathological ventricular remodelling is associated with distinct functional, cellular, histological, and molecular phenotypes. Proteomic profiling identified numerous proteins and processes that are distinctly altered in IGF1R Tg and DCM-dnPI3K Tg atria, as well as co-identification of proteins that are dysregulated in the pathological model (DCM-dnPI3K) and within the atria of humans with AF. Our open access proteomic data set provides a resource for the investigation of new protein targets and testing of potential therapeutic approaches for the prevention and treatment of adverse atrial remodelling and progression to AF.

## Clinical perspectives

- AF, the most common cardiac rhythm disorder worldwide, is associated with increased risk of HF and stroke. Earlier detection and new treatment strategies are greatly needed. Atrial enlargement in a cardiac disease setting is a key factor that predisposes to AF. In contrast, atrial enlargement in a physiological setting (e.g. exercise) does not typically progress to AF. A greater understanding of the molecular mechanisms underlying pathological and physiological atrial enlargement has the potential to uncover new therapeutic targets and biomarkers for AF.
- In the present study, we identified distinct functional, histological and molecular differences in atria from mice with pathological and physiological atrial enlargement. We also identified distinct proteins and signalling networks in the atria of the two models, many with known relevance to human AF.
- The proteomic resource will allow other investigators to study potential new regulators of atrial biology and function, drug targets and biomarkers for AF. Collectively, this has the potential to lead to better outcomes for patients with AF.

## Supplementary Material

Supplementary Figures S1-S5 and Tables S1-S15

## Data Availability

All supporting data are included within the main article and its supplementary files. MS-based proteomics data is deposited to the ProteomeXchange Consortium via the MASSive partner repository and available via MASSive with identifier (MSV000093605).

## Abbreviations

AF: atrial fibrillation
ANP: atrial natriuretic peptide
dnPI3K: dominant-negative PI3K
HF: heart failure
LA: left atrium
LAA: left atrium and appendage
LV: left ventricular
PI3K: phosphoinositide 3-kinase
PI3K (p110α): phosphoinositide 3-kinase, p110α isoform
RA: right atria
SEM: standard error of the mean
