# Supplementary material for: Distinct functional and molecular profiles between physiological and pathological atrial enlargement offer potential new therapeutic opportunities for atrial fibrillation
Source: Clin Sci (Lond). 2024 Jul 30;138(15):941–62. doi: 10.1042/CS20240178 (PMC11292366; doi:10.1042/CS20240178)
Supplement: Supplementary Figures S1-S5 and Tables S1-S15 [file CS-2024-0178_supp.zip › CS-2024-0178_suppsm.pdf]

Supplementary Material

Distinct functional and molecular profiles between physiological and pathological atrial enlargement offer potential new therapeutic opportunities for atrial fibrillation

Yi Ching Chen[1,2,3,4], Seka Wijekoon[1], Aya Matsumoto[1], Jieting Luo[1], Helen Kiriazis[1,3], Emma Masterman[1], Gunes Yildiz[1], Jonathon Cross[1], Adam C Parslow [1,3,4], Roger Chooi[1], Junichi Sadoshima[5]. David W Greening[1,3, 4], Kate L Weeks[1,6*] and Julie R McMullen[1,2,3,4,7*]

[1] Baker Heart and Diabetes Institute, Melbourne, Victoria, Australia

[2] Department of Diabetes, Central Clinical School, Monash University, Clayton, Victoria, Australia

[3] Baker Department of Cardiometabolic Health, The University of Melbourne, Melbourne, Victoria, Australia

[4] Baker Department of Cardiovascular Research, Translation and Implementation, La Trobe University, Melbourne, Victoria, Australia

[5] Department of Cell Biology and Molecular Medicine, Rutgers New Jersey Medical School

[6] Department of Anatomy and Physiology, University of Melbourne, Melbourne, Victoria, Australia

[7] Monash Alfred Baker Centre for Cardiovascular Research, Monash University, Melbourne, Victoria, Australia

* Joint senior authors

Correspondence:
Julie R McMullen
Baker Heart and Diabetes Institute
Julie.mcmullen@baker.edu.au

Kate Weeks
University of Melbourne
Kate.weeks@unimelb.edu.au

Yi Ching Chen
Baker Heart and Diabetes Institute
Peggy.chen@baker.edu.au

Contents:

Supplementary Table 1: List of Primers for Genotyping

Supplementary Table 2: List of Taqman Assays for mRNA Expression Analysis

Supplementary Figure 1: Experimental Cohort Details

Supplementary Figure 2: High magnification representative histological images of sections of the left atria in female mice with physiological or pathological atrial enlargement.

Supplementary Figure 3: Characterisation of left atrial morphology, function, and gene expression in 8-week-old female mice with physiological and pathological atrial enlargement.

Supplementary Figure 4: Principal component analysis (PCA) of Log2 intensity-transformed values of proteomics data from 8-week-old female mouse models.

Supplementary Figure 5: Regulation of proteins in LA from the physiological (IGF1R) and pathological model (DCM-dnPI3K).

Supplementary Table 1: List of Primers for Genotyping

Mouse strain	Primer Name	Primer Sequences (5' – 3')	Amplicon length (bp)
IGF1R Tg mice (tail clips)	IGF1R	Fwd: GGC ACT TTA CAT GGA GTC CT<br>Rev: GAA CAG CAG CAA GTA CTC GGT AAT	400
DCM-dnPI3K Tg mice (tail clips)	Mst1	Fwd: GGC ACT CTT AGC AAA CCT CA<br>Rev: GAT TCC ACA GGA ACT TGC TT	420
	dnPI3K	Fwd: GGC ACT TTA CAT GGA GTC CT<br>Rev: TGG CCT CTC TGA ACA GTT CAT	360

Supplementary Table 2: List of Taqman Assays for mRNA Expression Analysis

Gene	TaqMan® Gene Expression Assay	Amplicon Length (bp)	Exon Spanning	Dye	RefSeq
Natriuretic peptide type B; BNP *(Nppb)*	Mm01255770_g1	68	Yes	FAM	NM_001287348.1<br>NM_008726.5
ATPase, Ca++ transporting, cardiac muscle, slow twitch 2; SERCA2a *(Atp2a2)*	Mm01201431_m1	90	Yes	FAM	NM_001110140.3<br>NM_009722.3<br>NR_027838.1
Collagen, type I, alpha 1; Collagen 1 *(Col1a1)*	Mm00801666_g1	89	Yes	FAM	NM_007742.3
Toll-like receptor 4 (*Tlr4*)	Mm00445273_m1	87	Yes	FAM	NM_021297.2
Transcription Factor A, Mitochondrial *(TFAM)*	Mm00447485_m1	81	Yes	FAM	NM_009360.4
Hypoxanthine guanine phosphoriboxyl transferase; *Hprt1*	Mm01545399_m1	81	Yes	VIC	NM_013556.2

Supplementary Figure 1

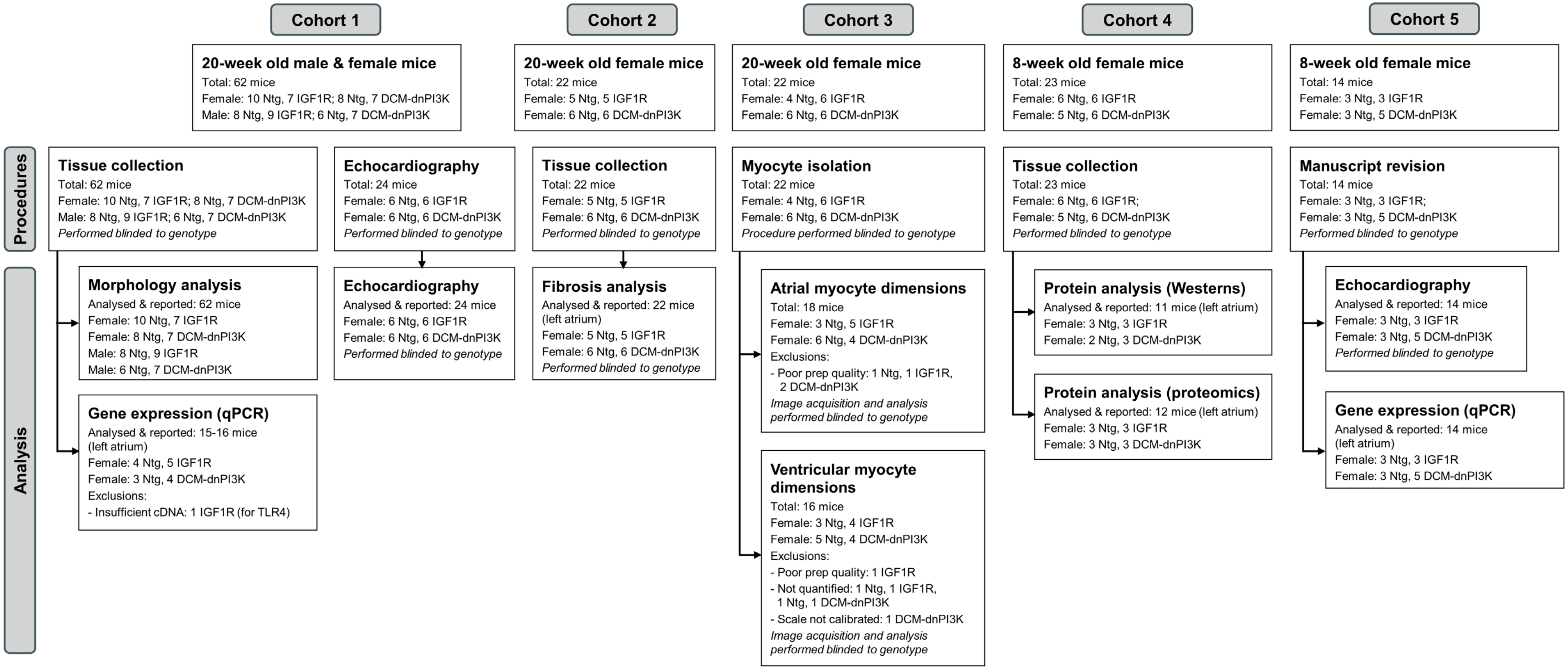

Supplementary Figure 1: Experimental Cohort Details. Flowchart showing sample size, sex, genotype, age and any exclusions for each experimental cohort and type of analysis performed.

Supplementary Figure 2

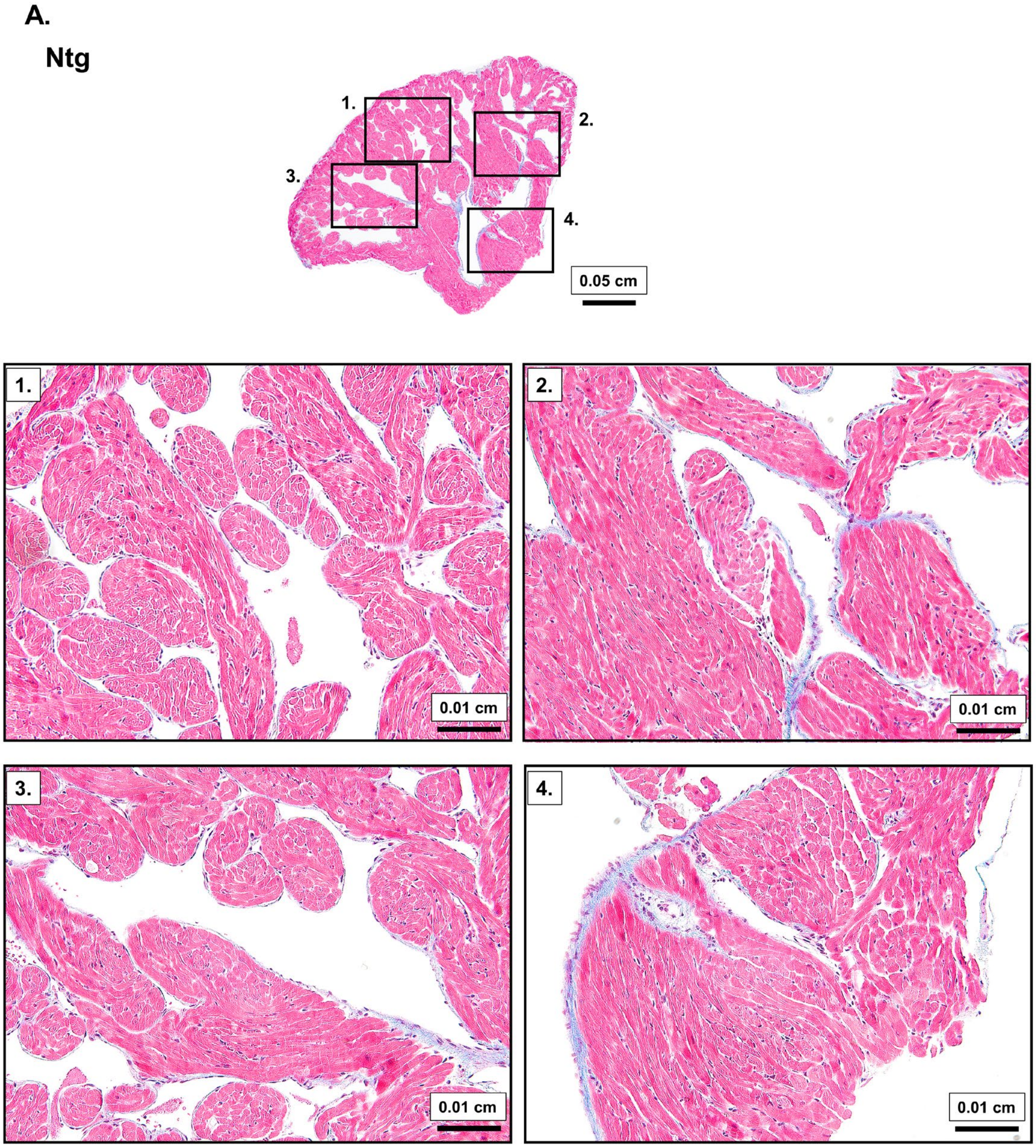

B.

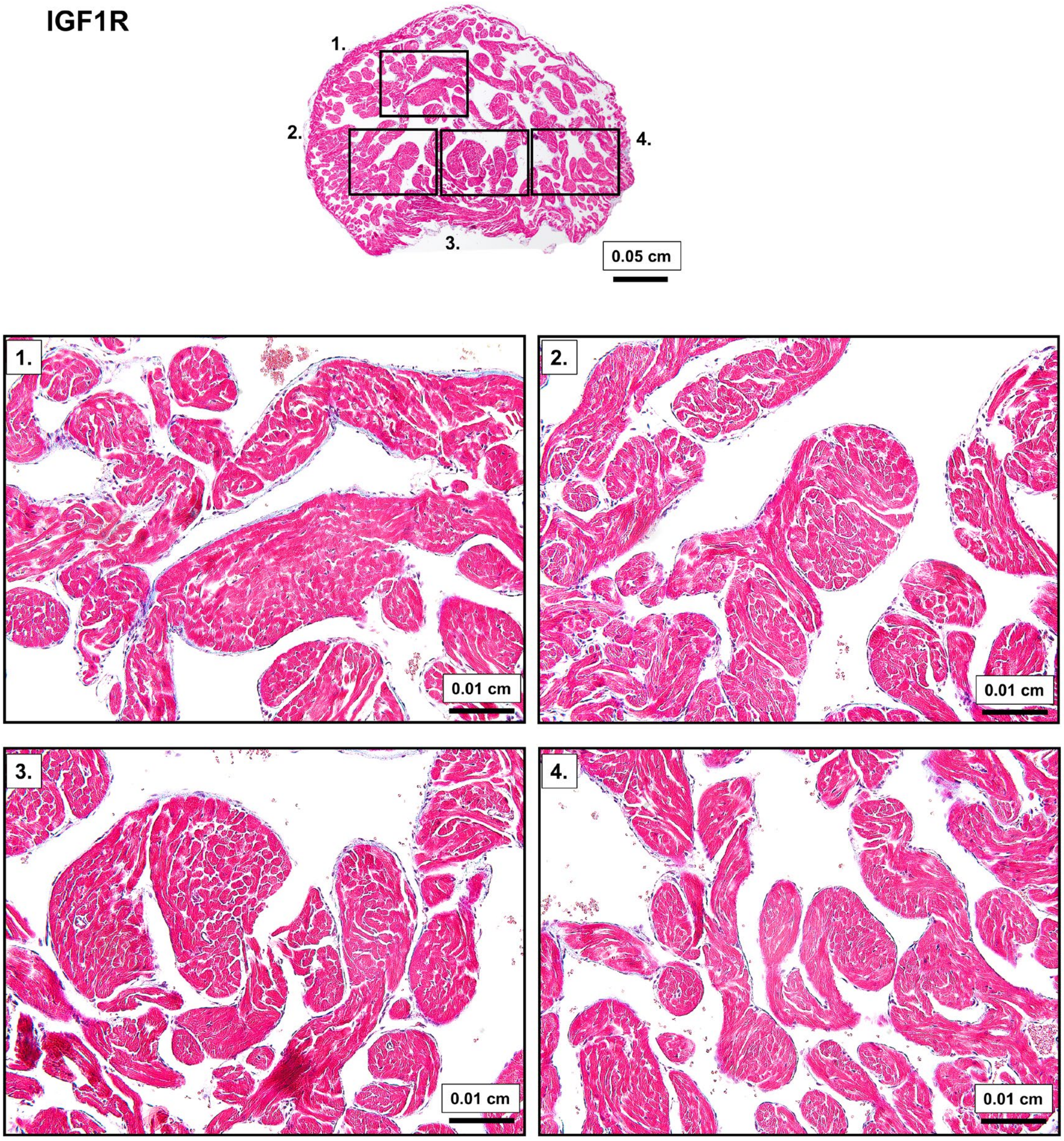

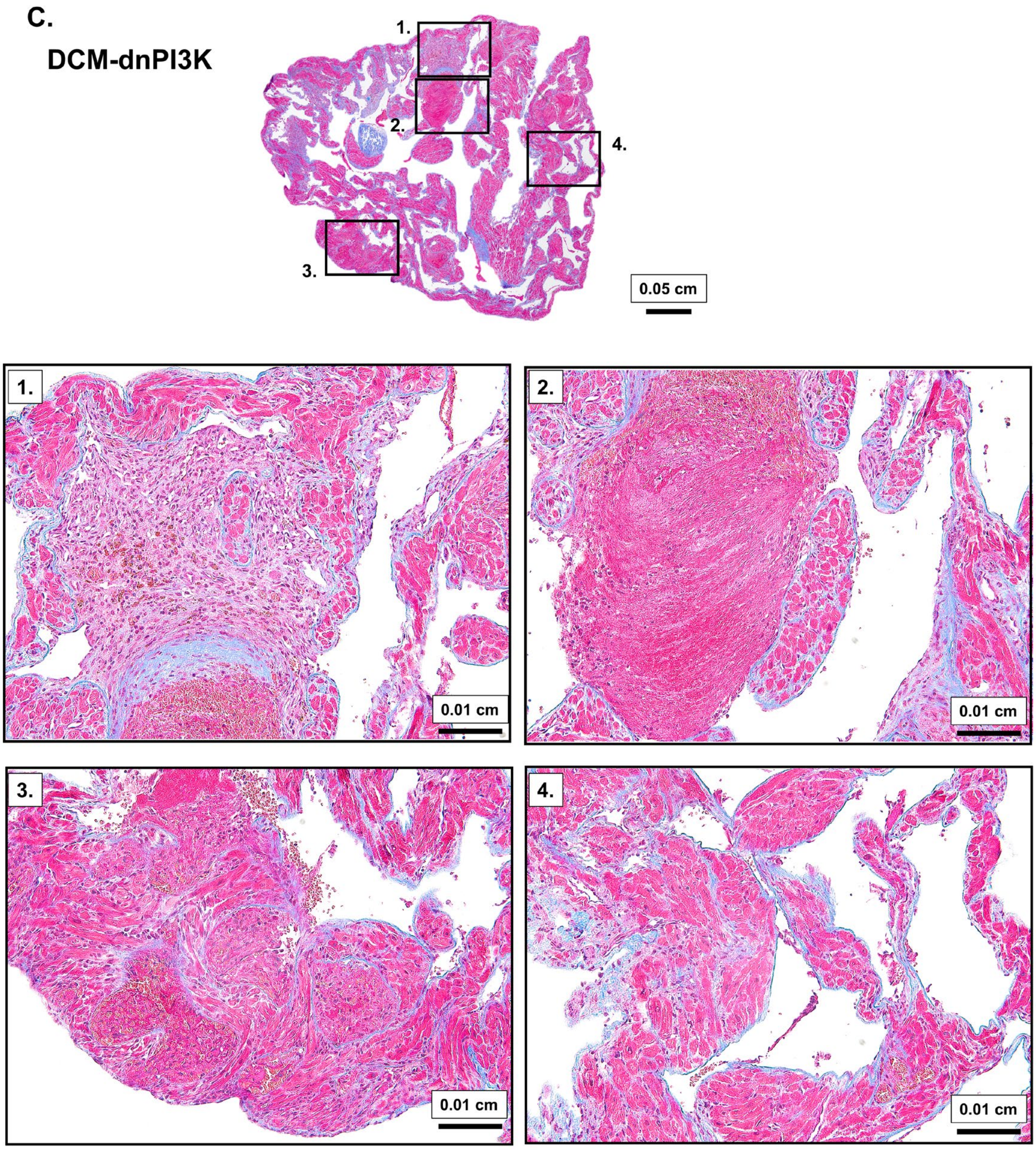

Supplementary Figure 2: High magnification representative histological images of sections of the left atria in female mice with physiological or pathological atrial enlargement. Histological examination of LA sections stained with Masson's Trichrome from 20-week-old female mice: non-transgenic (Ntg, **A**), physiological model (IGF1R, **B**), and pathological model (DCM-dnPI3K, **C**).

Supplementary Figure 3

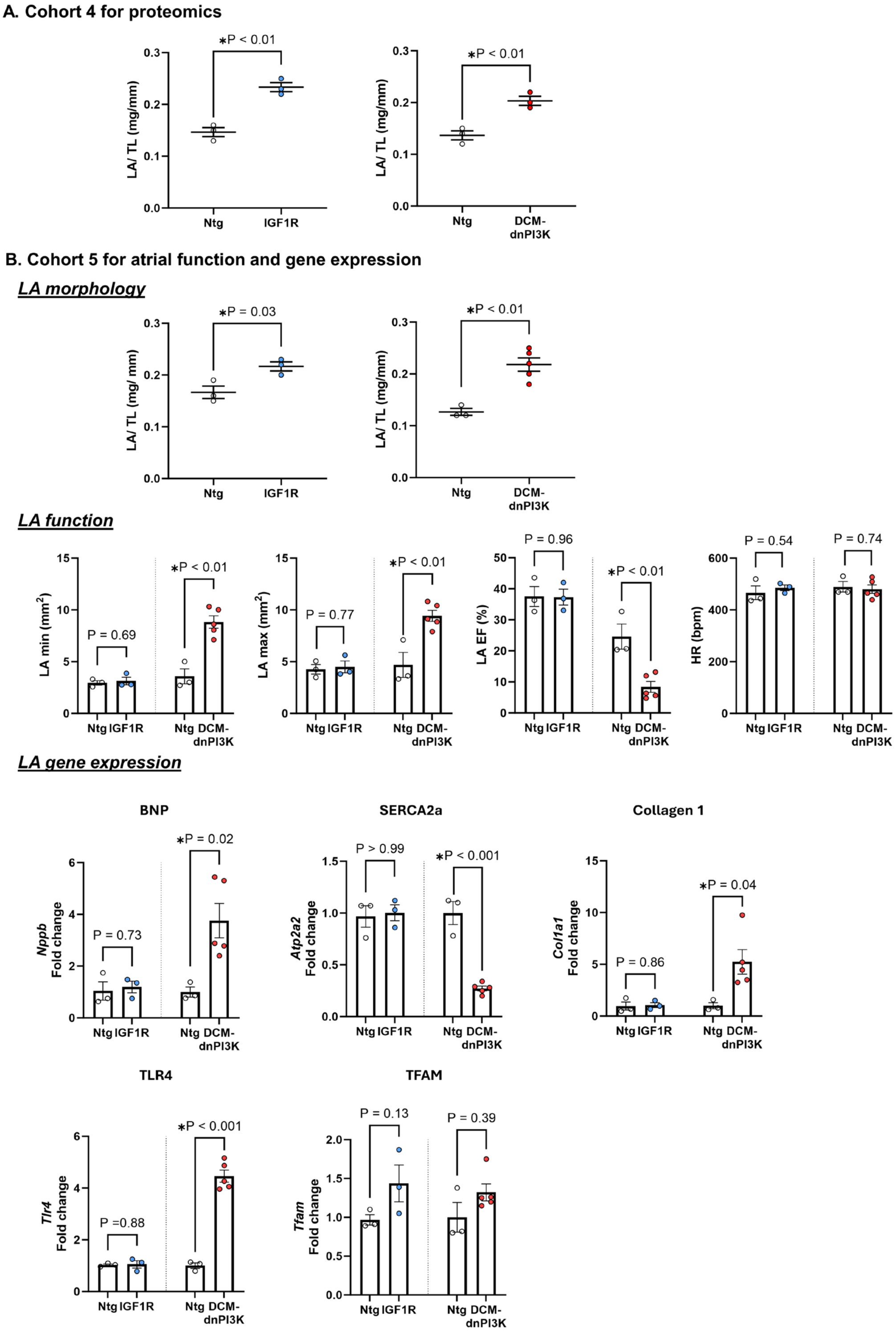

Supplementary Figure 3: Characterisation of left atrial morphology, function, and gene expression in 8-week-old female mice with physiological and pathological atrial enlargement. Left atrial (LA) weight normalized to tibia length (TL) in 8-week-old female mouse models with physiological (IGF1R Tg) and pathological cardiac enlargement (DCM-dnPI3K Tg) compared to non-transgenic (Ntg) controls for cohort 4 (used for proteomics, **A**) and cohort 5 (for LA function and gene expression assessments, **B**). Echocardiographic assessment of LA dimensions and function, including LA systolic area (LA min), LA diastolic area (LA max), and LA ejection fraction (LA EF). Heart rate

(HR) was comparable between groups. Gene expression analysis in the LA of 8-week-old female mice, assessed by qPCR. Analysed genes include B-type natriuretic peptide (BNP, Nppb), sarcoplasmic/endoplasmic reticulum calcium-ATPase 2a (SERCA2a, Atp2a2), collagen 1 (Col1a1), toll-like receptor 4 (Tlr4), and transcription factor A, mitochondrial (TFAM). Data are presented as mean ± SEM, (n=3-5/group). Unpaired t-tests were performed except for data that failed the normality test (SERCA2a Ntg vs IGF1R and TFAM Ntg vs DCM-dnPI3K), where a Mann–Whitney test was used.

Supplementary Figure 4

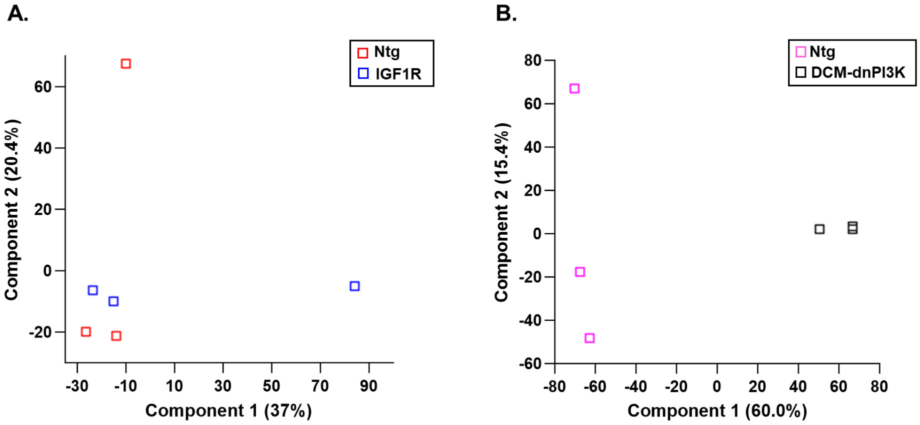

Supplementary Figure 4: Principal component analysis (PCA) of Log2 intensity-transformed values of proteomics data from 8-week-old female mouse models. PCA plot of valid values with a 70% cutoff in at least one group. The analysis is based on log2 intensity-transformed values of all samples. Missing values were replaced by imputation from a normal distribution (downshift 1.8, width 0.3, Perseus). **A.)** Physiological (IGF1R) and **B.)** pathological atrial enlargement (DCM-dnPI3K) compared to non-transgenic (Ntg) littermate controls.

Supplementary Figure 5

Ca^{2+} Handling / Myofibril assembly / Contractility:

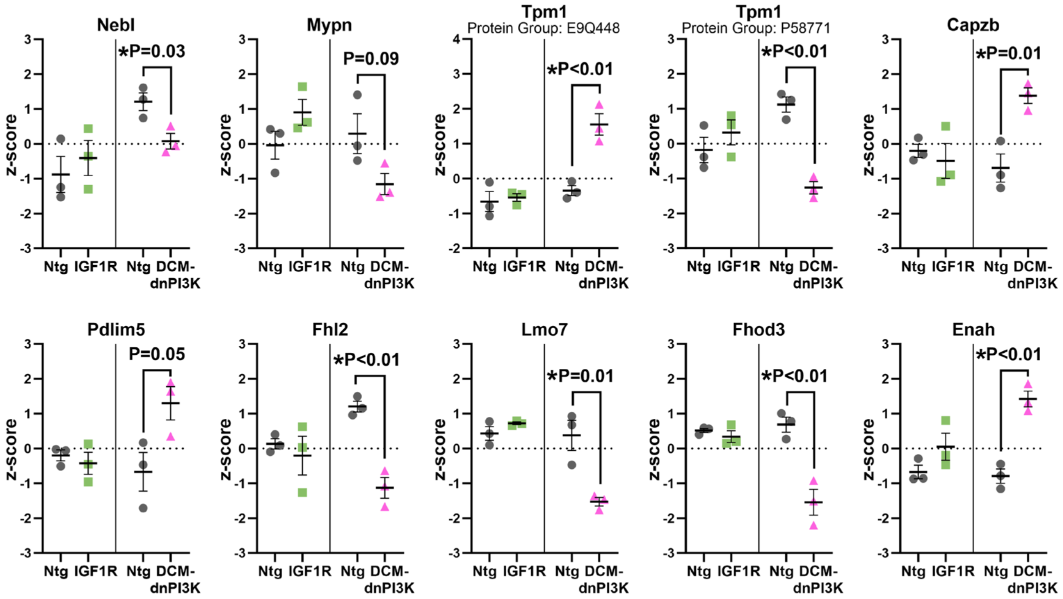

Metabolism / Mitochondria:

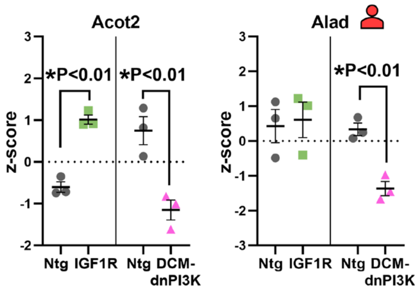

Extracellular matrix / Fibrosis:

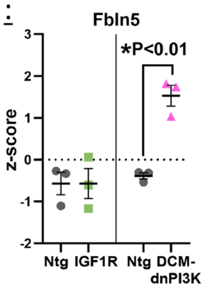

Supplementary Figure 5: Regulation of proteins in LA from the physiological (IGF1R) and pathological model (DCM-dnPI3K). Data points represent individual mice, presented as mean ± SEM. N=3/group (normalised intensity, z-score). Unpaired t-test. Upper red human torso highlights a protein co-identified in atrial tissue from patients with human AF. Protein names are defined in Supp Table 3.
