# Supplementary figures and images for: Distinct functional and molecular profiles between physiological and pathological atrial enlargement offer potential new therapeutic opportunities for atrial fibrillation
Source: Clin Sci (Lond). 2024 Jul 30;138(15):941–62. doi: 10.1042/CS20240178 (PMC11292366; doi:10.1042/CS20240178)

# **Uncropped Western Bolts for Figure 1**

Figure 1E

IGF1R

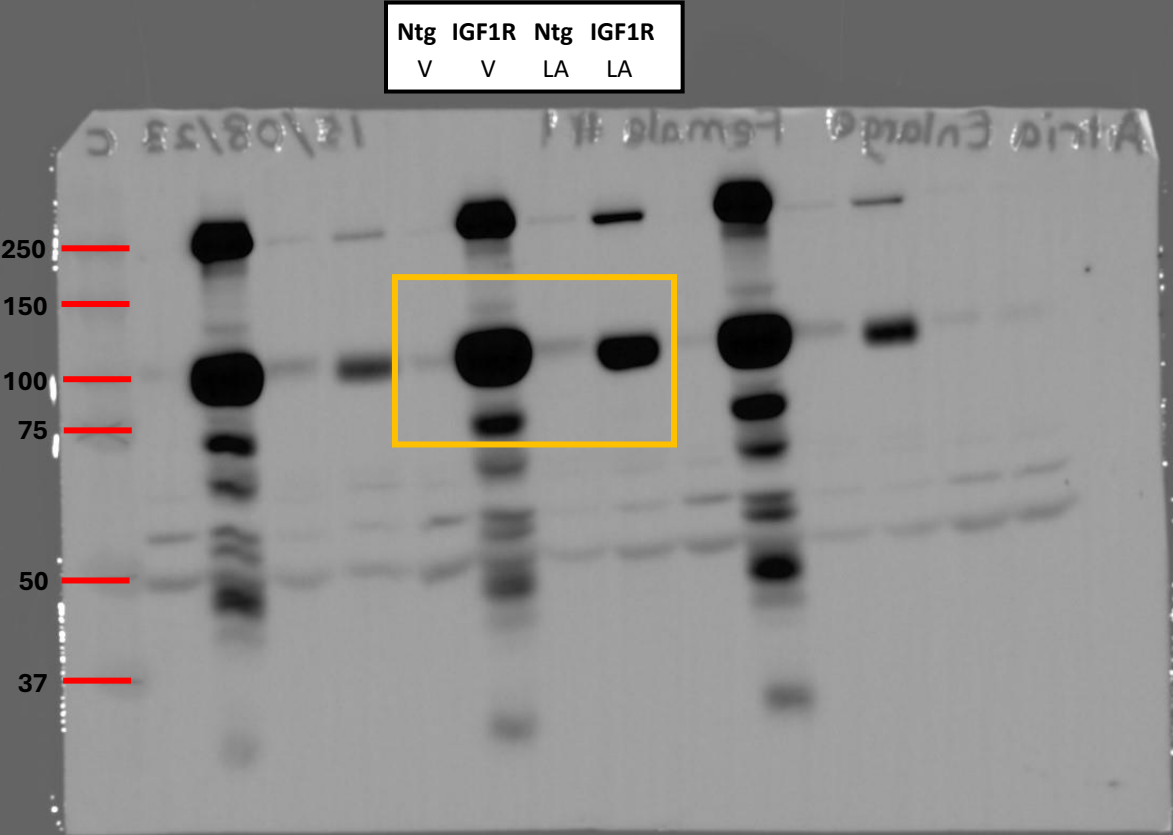

Figure 1E  
GAPDH

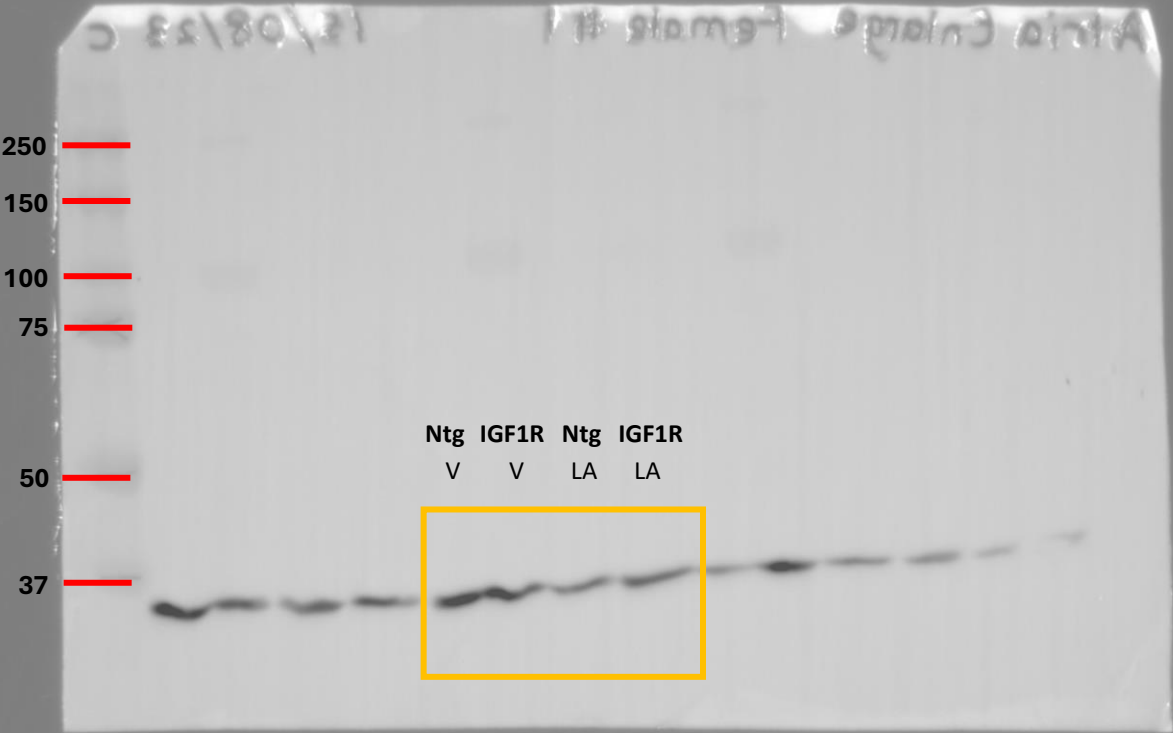

Figure 1F  
p110α

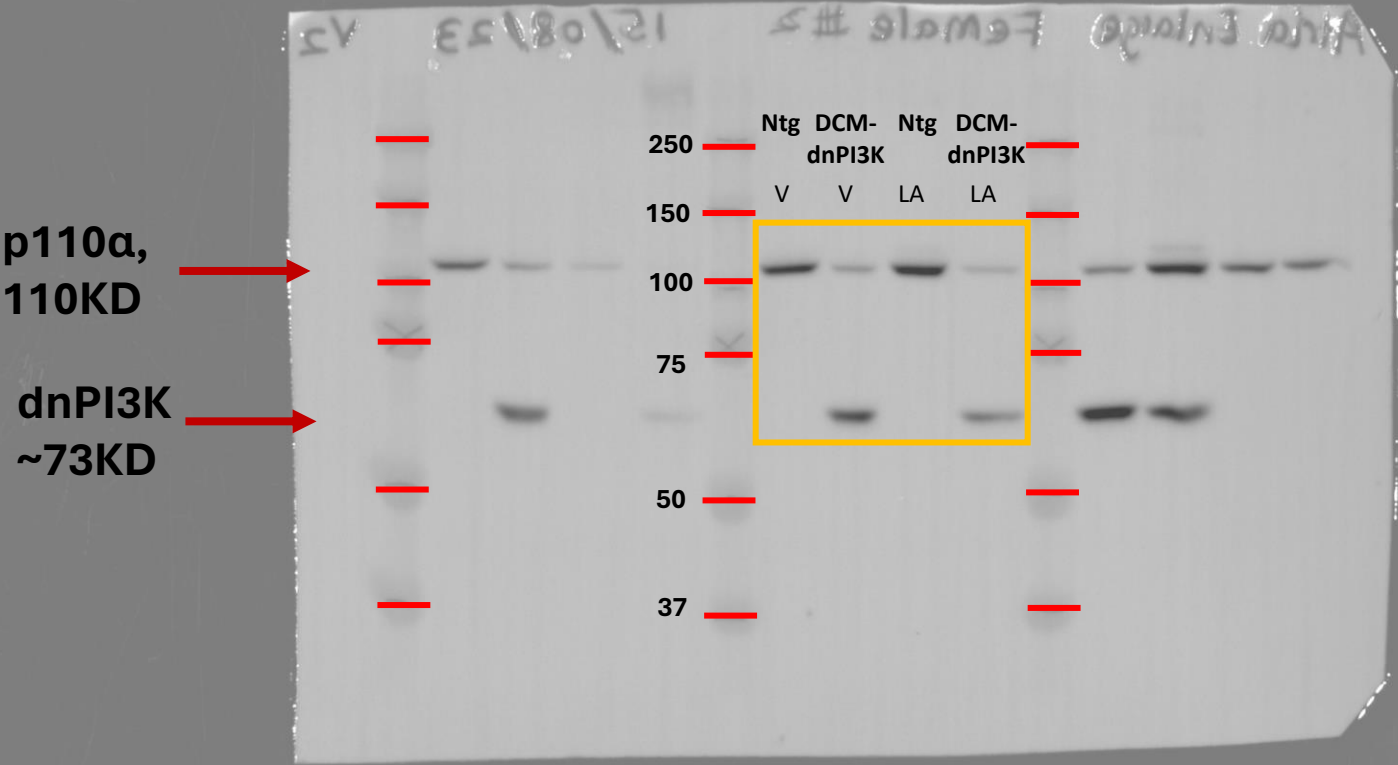

Figure 1F  
GAPDH

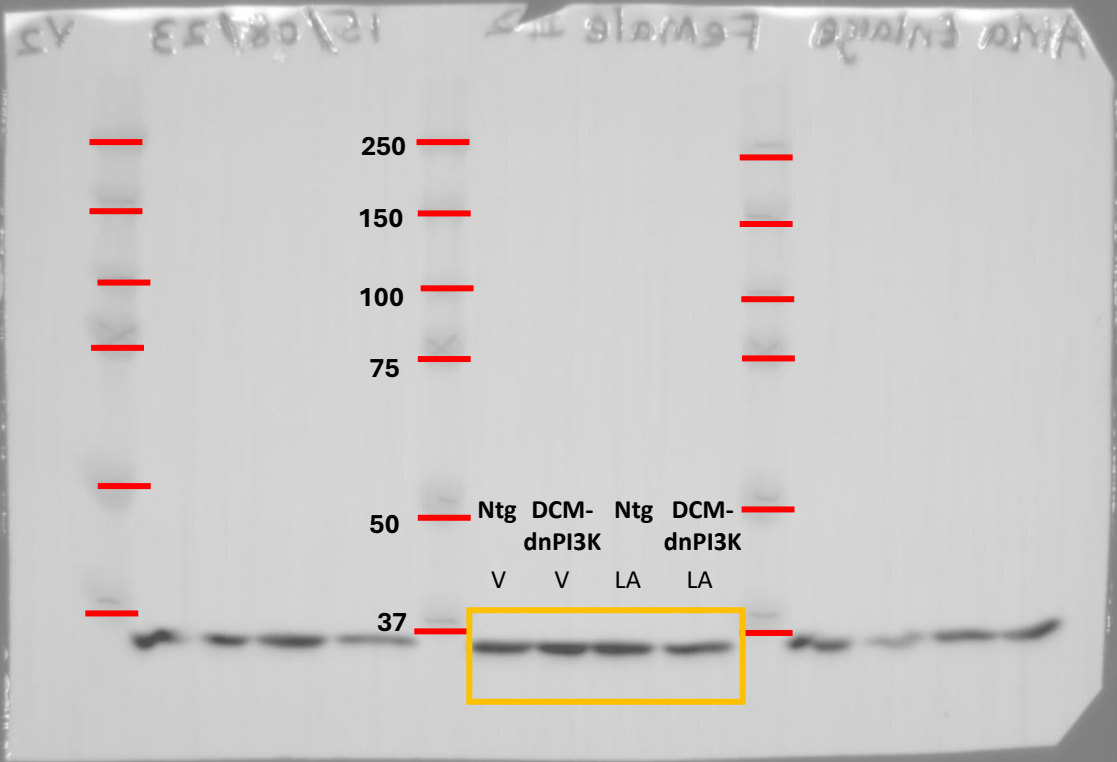

Supplement: Supplementary Figures S1-S5 and Tables S1-S15 [file CS-2024-0178_supp.zip › CS-2024-0178_suppwb.pdf]
